# The multifaceted roles and diagnostic-therapeutic potential of LINC01410 in malignant tumors and non-malignant disorders

**DOI:** 10.3389/fimmu.2025.1588816

**Published:** 2025-12-15

**Authors:** Kui Zhong, Qiang Yi, Xinting Ouyang, Weijian Zhu, Zheng Chen, Xinlin Wang, Yangneng Zeng, Jianing Zhong, Jinghua Zhong

**Affiliations:** 1 The First Clinical Medical College, Gannan Medical University, Ganzhou, Jiangxi, China; 2 School of Basic Medical Sciences, Gannan Medical University, Ganzhou, Jiangxi, China; 3 Department of Oncology, The First Affiliated Hospital of Gannan Medical University, Ganzhou, Jiangxi, China; 4 Jiangxi”Flagship” Oncology Department of Synergy for Chinese and Western Medicine, Ganzhou, Jiangxi, China; 5 Jiangxi Provincial Unit for Clinical Key Oncology Specialty Development, Ganzhou, Jiangxi, China; 6 Jiangxi Clinical Research Center for Cancer, Ganzhou, Jiangxi, China

**Keywords:** lncRNA, LINC01410, malignant tumors, non-malignant diseases, biology, clinical diagnosis and treatment

## Abstract

LINC01410 is a recently identified long non-coding RNA located on chromosome 9 that plays a multifaceted role in human diseases. Its dysregulation is closely associated with tumor initiation, progression, and therapeutic response. LINC01410 promotes oncogenesis by modulating key signaling pathways, such as PTEN/AKT, Notch, ErbB, and NF-κB, interacting with non-coding RNA networks, and influencing the expression of proteins involved in tumor biology. Beyond these roles, it contributes to cancer metabolism by regulating glycolysis, lipid droplet accumulation, and exosome-mediated intercellular communication, thereby shaping the tumor microenvironment and enhancing malignancy. Intriguingly, LINC01410 exhibits opposite expression patterns in non-malignant conditions such as preeclampsia and diabetic nephropathy, highlighting its context-dependent biological function. Collectively, these findings position LINC01410 as a pivotal regulator linking protein expression, metabolism, and intercellular signaling, underscoring its potential as a biomarker and therapeutic target in oncology.

## 1 Introduction

Cancer is a leading cause of death worldwide, second only to cardiovascular disease (1, 2), and it represents a terrible threat to the health of mankind. In recent years, malignant-tumor incidence and mortality rates have increased in all countries (3, 4). Given the absence of reliable biomarkers for early diagnosis, the majority of cancers advance to their later stages before detection. Consequently, a thorough understanding of the molecular mechanisms governing tumorigenesis is essential for the identification of robust early diagnostic markers and the development of effective therapeutic interventions.

As epigenetic regulators, LncRNAs are defined as non-coding transcripts exceeding 200 nucleotides in length. Many of these molecules, akin to mRNAs, undergo 5’ capping, splicing, and polyadenylation. LncRNAs are extensively involved in a wide array of biological and pathological processes (5). LncRNAs play a crucial role in the regulation of gene expression across multiple layers, including transcription, post-transcription, translation, and even post-translation. It is widely recognized that they contribute to the epigenetic modifications of chromatin (6). Notably, advancements in sequencing technologies and large-scale genomic projects have demonstrated that lncRNAs (exceeding 200 nucleotides in length) serve as critical regulatory factors in numerous human diseases, particularly cancer (7–9). LncRNAs engage in a wide array of physiological and pathological processes at the cellular level through diverse regulatory mechanisms. These encompass critical biological processes in cancer progression, such as proliferation, differentiation, stemness, migration, invasion, and apoptosis (10).

LINC01410 is a newly identified LncRNA, comprising 2,877 nucleotides. It is located on chromosome 9 (positions 62,801,461 to 62,813,486), a newly identified lncRNA (11). The altered expression of LINC01410 is closely associated with various clinicopathological characteristics and poor prognosis in cancer. Numerous lncRNAs function as competitive endogenous RNAs (ceRNAs), also referred to as miRNA sponges, regulating gene expression. These molecules are intimately linked to the proliferation, apoptosis, and migration of tumor cells (12–14). LINC01410 is upregulated in 15 types of cancer and inhibits the expression of 12 distinct miRNAs(as shown in **Table 1**). LINC01410 also plays a critical regulatory role in the pathogenesis and progression of certain non-malignant diseases, such as preeclampsia and diabetic nephropathy (11, 30). LINC01410 is involved in the regulation of four distinct signaling pathways that influence cancer progression. The expression levels of LINC01410 are closely associated with the drug sensitivity of glioblastoma and the radiosensitivity of neuroblastoma. LINC01410 is implicated in the glycolytic pathway, with aberrant expression influencing the progression of esophageal cancer and neuroblastoma. Exosome-derived LINC01410 modulates gene expression in recipient cells, playing a pivotal role in cancer development. For instance, exosome-derived LINC01410 has been shown to promote the progression of esophageal cancer. LINC01410 is engaged in the regulation of multiple protein levels and exerts a crucial influence in the progression of cancer.

**Table 1:** The role of LINC01410 in various cancers.

Tumor types.	Samples	Cell lines	Animals	Exp	Mechanism	EC	IC	Ref
OS	30 pairs of tissues	u-2os,Hf-oB1.19,sao-2,Hos,MG-63,143B	–	Up	LINC01410/miR- 122–5p/NDRG3	Proliferatio ↑ Invasion ↑ Migration ↑	–	(15)
	22	u-2os,Hf-oB1.19,sAos-2,Hos,MG-63	–	Up	LINC01410/miR- 3218	Invasion ↑ Growth ↑	–	(16)
	50	u-2os,Hf-oB1.19,sAos-2,Hos,MG-63,143B	–	Up	LINC01410/miR- 497–5p/HMGA2	Growth ↑ Invasion ↑ Migration ↑	–	(17)
BC	60	T24,J82, UMUC35637, SV-HU-1	Xenografted nude mice	Up	LINC01410/miR- 4319/snail1	Proliferatio ↑ Invasion ↑ Migration ↑ EMT ↑	Growth↑	(18)
TC	–	Nthy-ori3-1,TPC,BHP5-16,BHP2-7,K1	–	Up	LINC01410/miR- 3619–5p/FOXM1	Proliferatio ↑ Apoptosis ↓	–	(19)
CC	51	Normal cell lines and Normal(Ect1/E6E7)Hela,siHa	–	Up	LINC01410/miR- 2467–3p/VOPP1	Proliferatio ↑ Invasion ↑ Migration ↑	–	(20)
	25	Hela, siHa, Ms751	Female BABL/c nude mice	Up	LINC01410/miR- 532–5p/FASN	–	Growth ↑ LNM ↑	(21)
EC	587	RL95-2,HEC-1-A,KLE	–	Up	LINC01410/miR- 23C/CDH7	Proliferatio ↑ Invasion ↑ Migration ↑	–	(22)
ES CC	6	TE-1, Eca-109	BALB/C Nude mice.	Up	LINC01410/miR-122-5p/PKM2	Migration ↑ EMT ↑ Invasion ↑	Growth ↑	(23)
CCA	100	CCLP1,HuccT1,HuH-28,RBE,QBC939,HIBEC	–	Up	LINC01410/miR-124-3P/SMAD5	Proliferatio ↑ Invasion ↑ Migration ↑	–	(24)
NB	61	SK-N-SH,IMR-32,KeIIy,SH-SY5Y	Male BALB/C Nude mice.	Up	LINC01410/miR-506-3P/WEE1	Apoptosis ↓ Cycle ↓ Proliferatio ↑	–	(25)
	30	HEK293,SK-N-BE (2),,G1-L1-N	BALB/L Nude mice.	Up	LINC01410/miR-545-3P/HK2	RS ↓ Proliferatio ↑ Invasion ↑	–	(26)
CRC	53	HT-29,HCT116, SW620,LOVO	–	Up	LINC01410/miR-3218	Proliferatio ↑ Invasion ↑	–	(27)
GBM	75	LN-229(CRL-2611), T98G(CRL-1690), HEB,U251(CL-0237), SHG-44(CL-0207)	–	Up	LINC01410/miR-370-3P/PTEN/AKT	DS ↓ Apoptosis ↓	–	(28)
	–	SHG44,T98G,LN229, A172,HEB,HEK-293T	–	Up	LINC01410/miR-506-3P/NOTCH2/NOtch Signaling pathway	Proliferatio ↑ Apoptosis ↓	–	(29)

OS, osteosarcoma; BC, bladder cancer; TC, thyroid cancer; CC, cervical cancer; EC, endometrial cancer;ESCC,esophageal squamous cell carcinoma; GBM, glioblastoma; CCA,cholangiocarcinoma; NB, neuroblastoma; CRC, colorectal cancer; GBC, gallbladder cancer; EMT, epithelial-mesenchymal transition; ↑, promote; ↓, inhibit; Up, Upregul; DS, Drug sensitivity; RS, Radiation sensitivity; IC, Intracellular; EC, Extracellular; Exp, Expression.

This review highlights the latest discoveries regarding the roles of LINC01410 in human cancers and certain non-malignant diseases. We first summarize its clinical significance and expression patterns across diverse malignancies, followed by an in-depth discussion of its molecular mechanisms, including its involvement in radio-sensitivity, drug resistance, glycolysis, lipid metabolism, and protein regulation, as well as the influence of exosomal lncRNAs derived from cancer-associated fibroblasts. Furthermore, we delineate the signaling pathways associated with LINC01410 and emphasize its emerging roles in non-malignant disorders such as diabetic nephropathy. Through this comprehensive overview, we aim to deepen the understanding of the multifaceted oncogenic functions of LINC01410, underscore its potential as a prognostic biomarker and therapeutic target, and outline the current challenges and future directions in this evolving research field.

## 2 Clinical significance of LINC01410 in human malignancies: expression profiles and circulating biomarker potential

**Figure 1:**
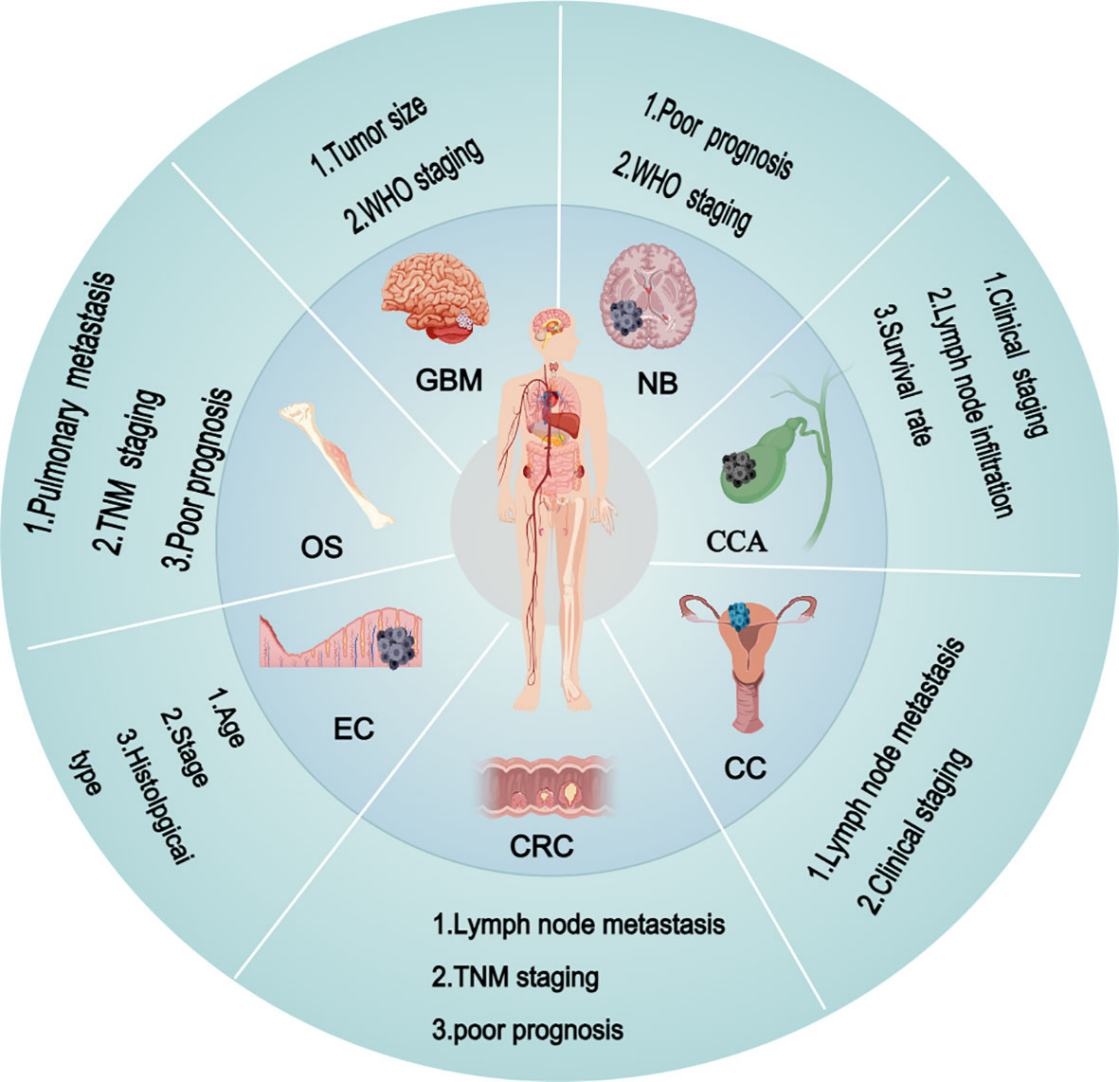
LINC01410 is significantly overexpressed in multiple types of malignancies, and its expression levels are closely associated with various clinicopathological features of these cancers. OS, osteosarcoma; CC, cervical carcinoma; EC, endometrial carcinoma; GBM, glioblastoma; CCA, cholangiocarcinoma; CRC, colorectal cancer; NB, neuroblastoma; WHO, World Health Organization; TNM, tumor–node–metastasis. This figure was created using Medpeer (www.medpeer.cn).

LINC01410 is notably overexpressed in various malignancies, including papillary thyroid carcinoma, endometrial carcinoma, cholangiocarcinoma, neuroblastoma, bladder cancer, gallbladder cancer, cervical carcinoma, and osteosarcoma. Moreover, its expression levels exhibit significant correlations with the clinical characteristics of these cancer**s** (as shown in **Figure 1**). This underscores the potential of LINC01410 as a pivotal biomarker, holding significant value for the prevention and treatment of cancer. For instance, in cervical carcinoma, elevated expression of LINC01410 is positively correlated with lymph node metastasis and clinical staging (20, 21). In gallbladder cancer, LINC01410 is associated with pathological staging, lymphatic invasion, and patient survival rates (31). Furthermore, in colorectal cancer, LINC01410 is linked to lymph node metastasis, TNM staging, and poor prognosis (32). In endometrial carcinoma, the overexpression of LINC01410 is strongly correlated with patient age, histological grade, histological subtype, clinical staging, and mortality (22). In osteosarcoma, LINC01410 overexpression is significantly associated with pulmonary metastasis, TNM staging, and poor prognosis (15). Additionally, in glioblastoma, the overexpression of LINC01410 is linked to tumor size and WHO grading (29). In neuroblastoma, the overexpression of LINC01410 is significantly correlated with adverse prognosis and WHO grading (25). Consequently, LINC01410 overexpression is closely associated with critical clinicopathological features, including tumor size, invasion depth, lymph node metastasis, and TNM staging. This underscores the potential of LINC01410 as a pivotal biomarker, holding significant value for the prevention and treatment of cancer.

Beyond its aberrant expression in tumor tissues, emerging evidence highlights the diagnostic and prognostic potential of circulating LINC01410 in peripheral blood. The detection of LINC01410 in serum and plasma samples offers a minimally invasive approach for cancer diagnosis, disease monitoring, and prognostic assessment. Recent studies in non–small cell lung cancer (NSCLC) and colorectal cancer (CRC) have provided compelling support for its clinical utility in the liquid biopsy setting (27, 33). Clinical analyses revealed that circulating LINC01410 is markedly elevated in patients with NSCLC relative to healthy controls. LINC01410 achieved the highest diagnostic accuracy for distinguishing metastatic from non-metastatic tumors (AUC = 0.77, 95% CI: 0.65–0.89) (33). Importantly, it outperformed the conventional marker CEA in both sensitivity and specificity and showed superior specificity for predicting metastatic risk (33).Although the oncogenic mechanisms of LINC01410 in NSCLC have not been fully elucidated, previous studies have provided insights into potential pathways. For instance, elevated AKT expression together with reduced PTEN expression has also been closely associated with NSCLC progression (33). In glioblastoma, high LINC01410 expression has been shown to correlate with malignant progression; its knockdown promotes apoptosis, enhances PTEN expression, and suppresses AKT phosphorylation (28). These findings suggest that LINC01410 may contribute to the pathological processes of NSCLC by modulating either the PTEN/AKT signaling pathway.

In addition to its remarkable clinical diagnostic and prognostic value in non-small cell lung cancer, circulating LINC01410 has also been reported to be upregulated in both colorectal cancer tissues and cell lines. Further experimental evidence demonstrated that LINC01410 suppresses the transition of the cell cycle from the G0/G1 phase to the S and G2/M phases, thereby inducing cell cycle arrest at G0/G1 (32). Moreover, compared with healthy controls and patients with colorectal polyps, circulating LINC01410 levels were found to be significantly elevated in individuals with colorectal cancer. Analysis of clinical data from 460 CRC patients in the TCGA database revealed that LINC01410 expression was significantly associated with TNM stage and lymph node metastasis (27). ROC curve analyses demonstrated that circulating LINC01410 effectively distinguished CRC patients from healthy controls, achieving superior sensitivity and AUC (0.894, 95% CI = 0.829–0.858) compared with conventional markers CEA and CA199 (27). These findings indicate that serum LINC01410 exhibits markedly higher diagnostic accuracy than traditional CRC biomarkers (27). Notably, logistic regression analysis demonstrated that combining LINC01410 with CEA and CA199 improved diagnostic performance for CRC patients, achieving an AUC of 0.892, higher than any single marker alone (27). These findings underscore the potential of circulating lncRNAs as robust biomarkers, and suggest that integrating additional lncRNAs or employing multi-marker models may further optimize their clinical utility (27).

In summary, LINC01410 is broadly overexpressed across various malignancies and closely associated with key clinicopathological features. Additionally, its circulating levels in peripheral blood hold significant diagnostic and prognostic value. These findings highlight the potential of LINC01410 as a biomarker bridging tissue and liquid biopsy, offering new avenues for early cancer detection and precision therapy.

## 3 The multifaceted oncogenic functions of LINC01410

LINC01410 exerts multifaceted oncogenic effects across various malignancies by regulating a wide range of biological processes. Through ceRNA-mediated mechanisms, it influences cell proliferation, migration, invasion, EMT, and apoptosis. Moreover, LINC01410 modulates radioresistance, chemoresistance, and metabolic reprogramming, including glycolysis and lipid metabolism, while facilitating intercellular communication via CAF-derived exosomes. Its dysregulated expression also alters multiple cancer-related proteins, collectively driving tumor progression. These findings underscore the broad oncogenic potential of LINC01410 and its promise as a diagnostic and therapeutic target in human cancers.

### 3.1 Mechanisms of LINC01410-mediated biological functions

**Figure 2:**
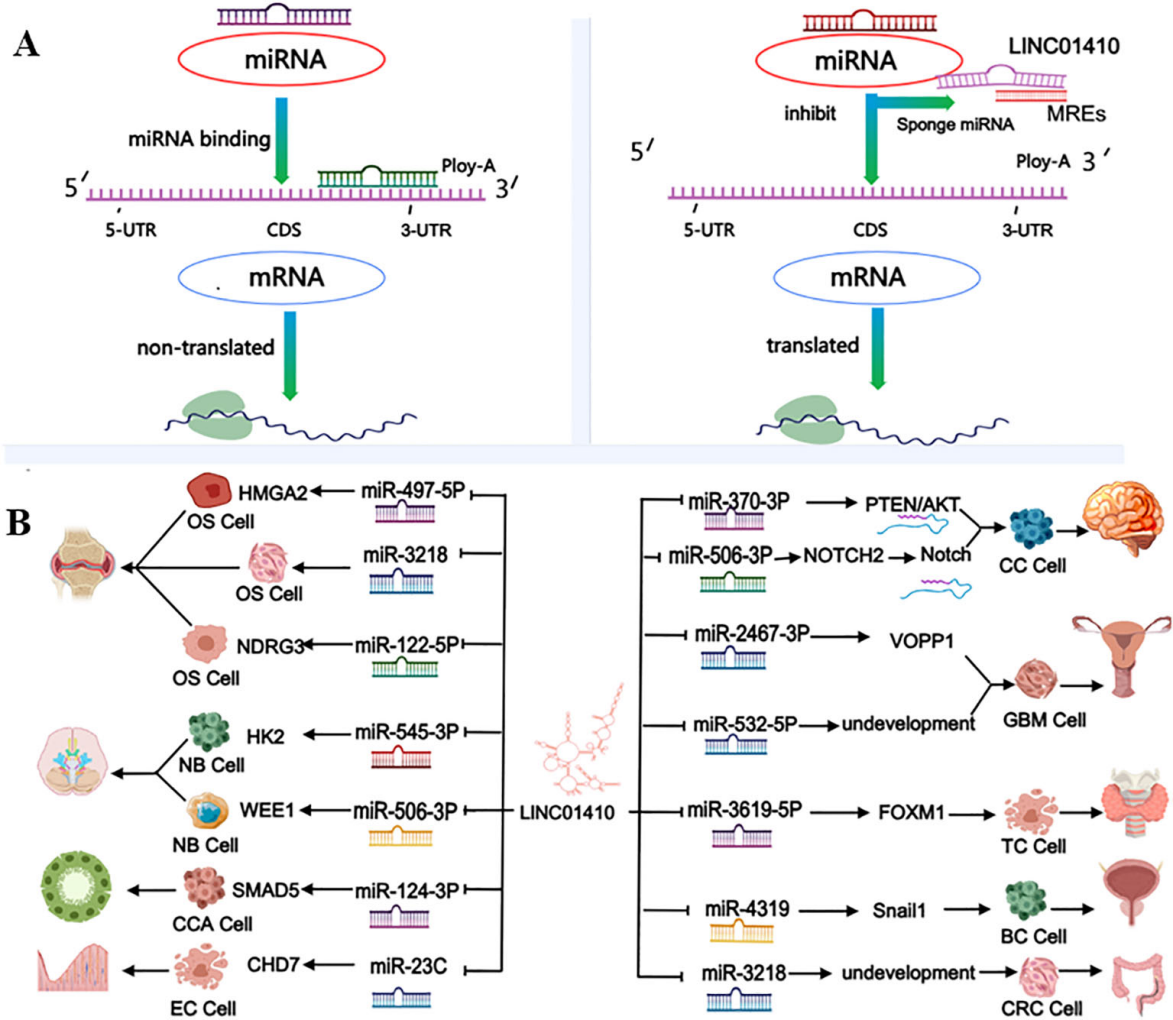
In **(A)**, Under physiological conditions, miRNAs bind to the 3′-UTR of target mRNAs, inducing translational repression or mRNA degradation. LINC01410 functions as a competing endogenous RNA (ceRNA), sequestering specific miRNAs via its miRNA response elements (MREs) and thereby preventing their interaction with target mRNAs. This alleviation of miRNA-mediated repression facilitates mRNA translation, highlighting a post-transcriptional mechanism by which LINC01410 modulates gene expression in cancer cells. In **(B)**, The LINC01410-centered ceRNA network involves 12 miRNAs across 10 types of cancer. LINC01410 can participate in cancer initiation and progression through interactions with multiple miRNAs. OS, osteosarcoma; CC, cervical cancer; TC, thyroid cancer; BC, bladder cancer; CC, colon cancer; GBM, glioblastoma; EC, endometrial cancer; CCA, cholangiocarcinoma; NB, neuroblastoma. This figure was created using Medpeer (www.medpeer.cn).

The ceRNA network centered around LINC01410 has unveiled a growing array of lncRNAs functioning as ceRNAs, thereby safeguarding mRNA from miRNA-mediated silencing (34). Non-coding RNAs (ncRNAs) have been defined as the new central dogma in cancer biology (7, 35). With the rapid advancements in the study of the interactive mechanisms of LncRNAs in tumor progression, it has become increasingly challenging to view the functions of LncRNAs in isolation. The ceRNA hypothesis has introduced an exciting new dimension to RNA biology (36, 37). In cancer, the ceRNA regulatory networks composed of lncRNAs play a pivotal role. The ceRNA network centered around LINC01410 involves 12 distinct miRNAs across 10 types of cancer, including miR-122-5p, miR-4319, miR-3619-5p, miR-2467-3p, miR-3218, miR-497-5p, miR-23c, miR-124-3p, miR-370-3p, miR-506-3p, miR-532-5p, and miR-545-3p(as shown in **Figure 2**). Moreover, aberrantly expressed LINC01410 exerts its oncogenic effects by regulating various cellular processes, including proliferation, migration, invasion, EMT, and apoptosis(as shown in **Table 1**). This review aims to elucidate the underlying mechanisms of the LINC01410-centered ceRNA network in the pathogenesis of various malignant tumors.

LINC01410 is markedly upregulated in osteosarcoma (OS) cells and tissues, where it downregulates miR-122-5p, miR-497-5p, and miR-3128 while upregulating the expression of NDRG3 and HMGA2, thereby promoting OS cell proliferation, invasion, and migration (15–17).In neuroblastoma, LINC01410 acts as a sponge for miR-506-3p and miR-545-3p, respectively upregulating WEE1 and HK2, thereby promoting cancer cell proliferation, invasion, and migration, inhibiting apoptosis and cell cycle progression, and reducing radiosensitivity (25, 26). In cervical cancer, LINC01410 can sponge miR-2467-3p and miR-532-5p, upregulating VOPP1 and promoting the accumulation of lipid droplets, which in turn enhances the proliferation, invasion, and migration of cervical cancer cells (20, 21) (as shown in **Table 1**).

LINC01410 can influence the development of glioma by sponging miR-506-3p. Experimental evidence has confirmed that MYC acts as a transcription factor for LINC01410 in glioma, where MYC stimulates the overexpression of LINC01410 (29). In esophageal cancer, LINC01410 acts as a ceRNA by sponging miR-122-5p, thereby upregulating PKM2, respectively, ultimately contributing to the progression.

Studies on cholangiocarcinoma (CCA) have demonstrated that LINC01410 acts as a sponge for miR-124-3p, upregulating SMAD5, thereby promoting the proliferation, invasion, and migration of CCA cells and tissues (24). In studies related to bladder cancer (BC), it has been confirmed that LINC01410 functions as a sponge for miR-4319, upregulating Snail1, thereby promoting the proliferation, migration, invasion, and epithelial-to-mesenchymal transition (EMT) of BC cells (18). Studies on thyroid cancer (TC) have shown that LINC01410 absorbs miR-3619-5p, thereby upregulating FOXM1, which promotes TC cell proliferation and delays TC cell apoptosis (19). However, FOXM1 can transcriptionally activate LINC01410 in the PTC cell line, forming a positive feedback loop of LINC01410/miR-3619-5p/FOXM1 that regulates PTC cell proliferation and apoptosis (19). Studies on endometrial carcinoma have shown that LINC01410 acts as a sponge for miR-23c, promoting the upregulation of CHD7, thereby enhancing the proliferation, invasion, and migration of EC cells (22). In esophageal cancer research, experiments have shown that LINC01410 indirectly upregulates the expression of PKM2 in ESCC cells and tissues by acting as a sponge for miR-122-5p, thereby promoting ESCC cells migration, invasion, and EMT (23). In conclusion, LINC01410 may serve as an effective therapeutic target in cancers such as cholangiocarcinoma and bladder cancer.

In glioblastoma, LINC01410 acts as a sponge for miR-506-3p, thereby promoting NOTCH2 expression and activating the Notch pathway, which stimulates GBM cell proliferation and inhibits apoptosis (29). However, silencing LINC 01410 can target miR-370-3p, leading to the inactivation of the PTEN/AKT pathway, which suppresses GBM cell viability, enhances sensitivity to chemotherapy, and accelerates cancer cell apoptosis (28) (as shown in **Table 1**).

### 3.2 The role of exosomal lncRNA secreted by CAFs in cancer progression

Regulatory genes within malignant tumor cells, due to expression dysregulation, either suppress or promote tumor cell proliferation, invasion, or metastasis. In fact, regarding the origin of tumor-associated regulatory genes, in addition to the intrinsic genes present within the tumor cells and tissues themselves, genes that are carried by exosomes and transferred into tumor cells also play a crucial role in cancer progression. Currently, activated cancer-associated fibroblasts (CAFs) are primarily believed to originate from fibroblasts in the surrounding tissue, which are educated by cancer cells (38). CAFs are the principal stromal cells within the tumor microenvironment, playing a pivotal role in tumor proliferation and metastasis through paracrine signaling processes (23). Studies have already demonstrated that exosomes, as mediators of intercellular communication, play a significant role in cancer therapy (39, 40). Growing evidence increasingly suggests that exosomes can promote tumor initiation, progression, and advancement (41). Zhi Huashi and colleagues investigated the mechanisms by which exosomes derived from CAFs deliver LINC01410 in esophageal squamous cell carcinoma (ESCC) cells. The results demonstrated that ESCC cells internalize exosomes secreted by CAFs. These CAF-derived exosomes exert their effects by transferring LINC01410 into ESCC cells, leading to a significant elevation in LINC01410 levels within the cells. LncRNAs can be protected by exosomes, safeguarding them from degradation in the circulation, thereby enabling their potential use in the early diagnosis of cancer (42). The results reveal that LINC01410 is significantly overexpressed in ESCC tumor tissues compared to adjacent normal tissues (23). We have confirmed that the overexpression of LINC01410 markedly enhances cell migration and invasion.

**Figure 3:**
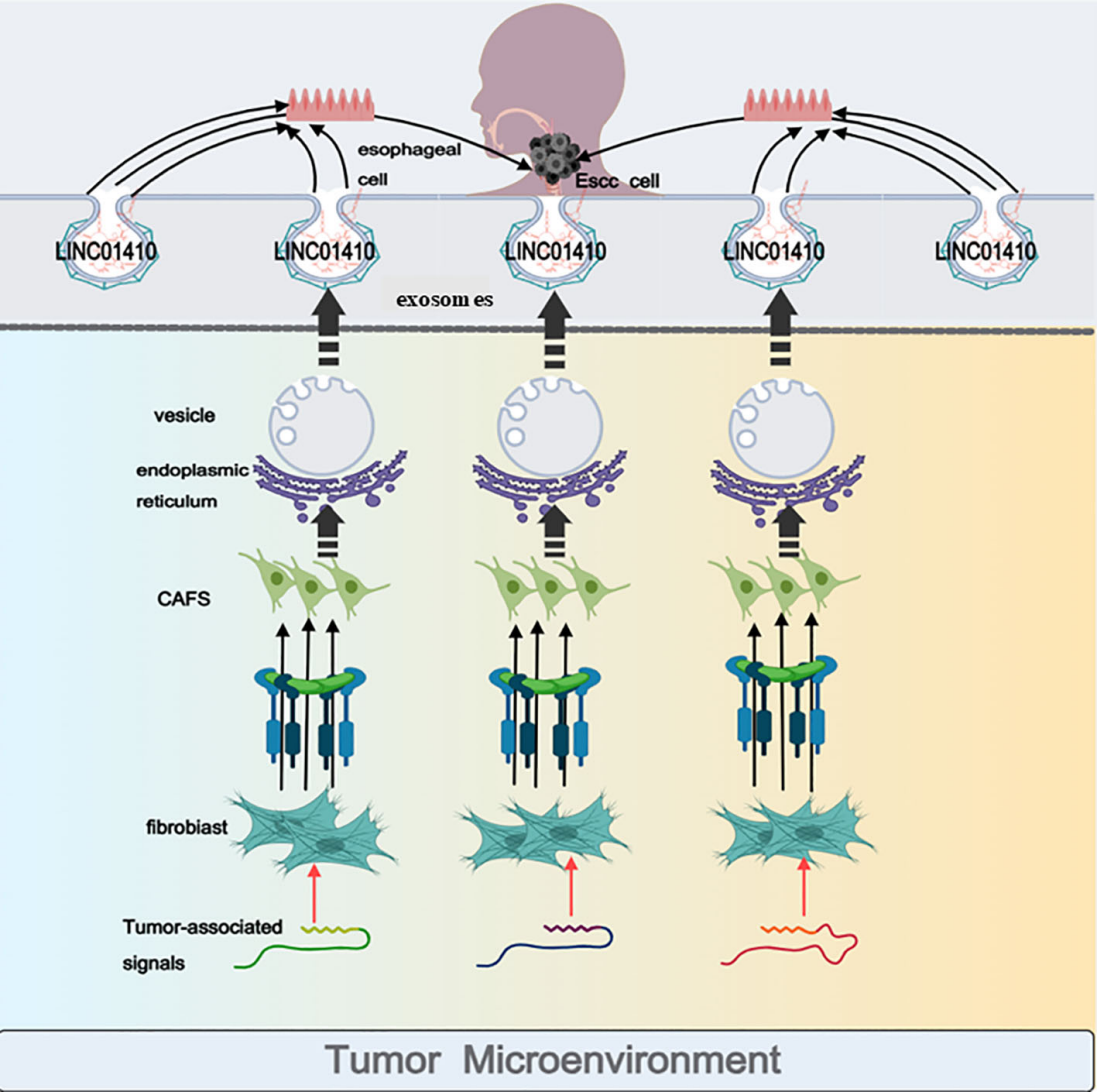
CAFs (stromal cells) secreted by fibroblasts are exocytosed upon entering the omentum. These CAFs secrete a substantial amount of LINC01410, which is transferred to normal esophageal cells. This process leads to a dramatic increase in LINC01410 levels within normal esophageal cells, subsequently contributing to the development of esophageal cancer. This figure was created using Medpeer.

In fact, exosome-mediated delivery of lncRNAs not only plays a pivotal role in the progression of ESCC but also exerts crucial regulatory functions in the initiation and development of other malignant tumors. Previous studies have demonstrated that in breast cancer cells, exosomal lncRNA SNHG3 secreted by CAFs acts as a molecular sponge for miR-330-5p (43). CAFs promote stemness and chemoresistance in colorectal cancer by transferring the exogenous lncRNA H19 (44). Thus, it is evident that exosomes secreted by CAFs serve as both bridges and hubs between cells, facilitating the transport of exogenous lncRNAs to normal tissues and cells, resulting in the dysregulation of endogenous lncRNAs and ultimately contributing to malignant transformation (as shown in **Figure 3**). In summary, both exosomes and LINC01410 hold great promise as pivotal therapeutic targets for the future treatment of ESCC. Inhibiting exosome secretion and intercellular transfer offers a valuable reference and novel approach for cancer prevention and therapy.

### 3.3 The role of LINC01410 in glycolysis

**Figure 4:**
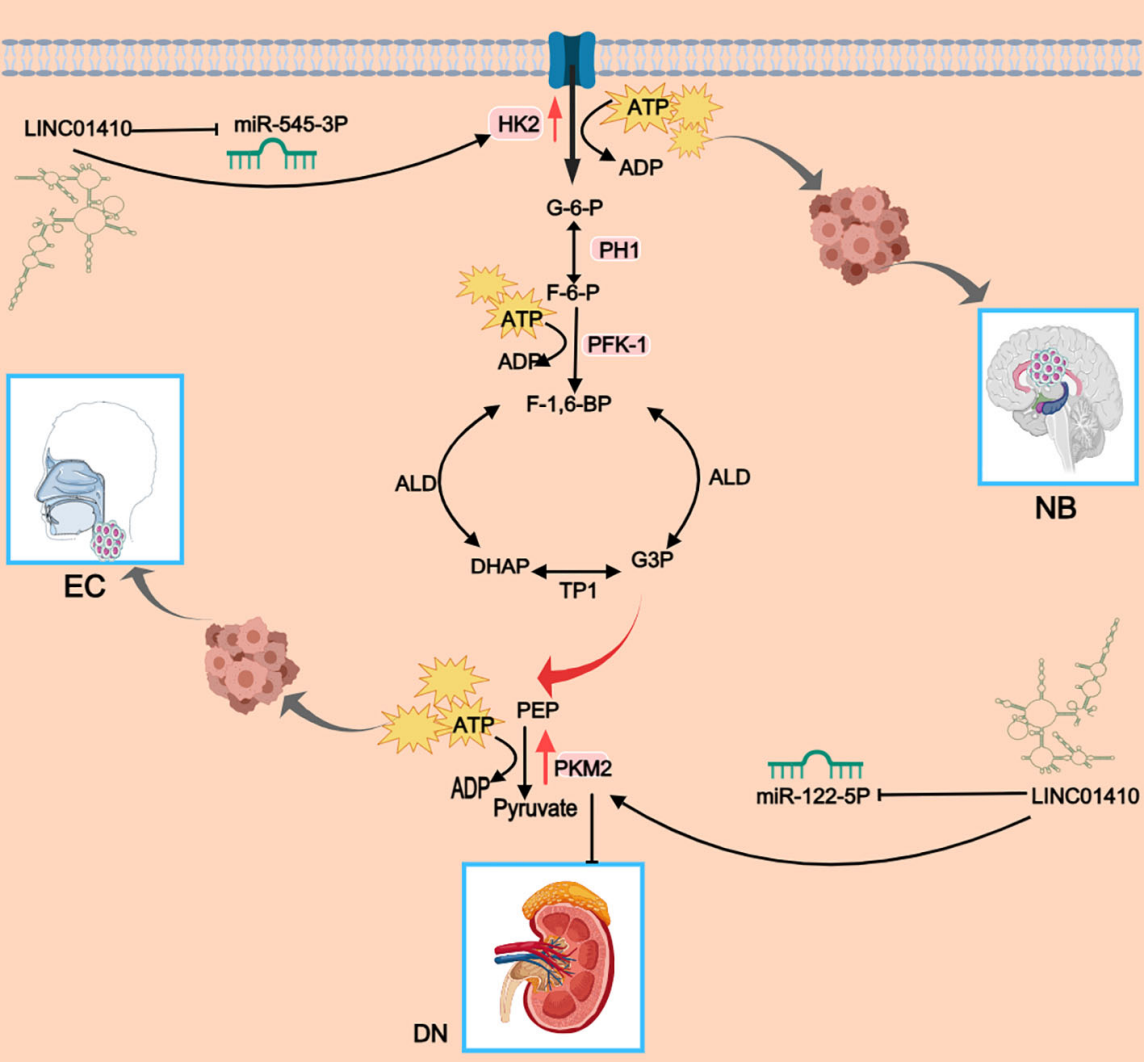
The fundamental distinction between tumor cells and normal cells lies in their metabolic patterns, with nearly 50% of ATP in tumor cells being generated through the glycolytic pathway. As key members of the hexokinase and pyruvate kinase families, respectively, HK2 and PKM2 play essential catalytic roles in glycolysis, producing ATP necessary for cancer cell survival and progression. Studies have shown that LINC01410 promotes the expression of its downstream targets HK2 and PKM2 by suppressing miR-545-3p and miR-122-5p, thereby accelerating the glycolytic process and generating ATP required for tumor cell survival and growth. This mechanism ultimately drives the tumorigenesis and progression of neuroblastoma and esophageal carcinoma. Interestingly, PKM2 has also been reported to enhance podocyte function and delay the progression of diabetic nephropathy (DN). In studies related to DN, LINC01410 improves podocyte function and mitigates disease progression by inhibiting miR-122-5p and consequently upregulating PKM2 expression. This figure was created using Medpeer.

The fundamental distinction between tumor cells and normal cells lies in their metabolic patterns (23). Nearly 50% of ATP in tumor cells is synthesized through the glycolytic pathway (23). This phenomenon, known as the Warburg effect, refers to the ability of malignant cells to obtain energy predominantly via glycolysis, producing lactate even under normoxic conditions (45). Increasing evidence has revealed that PKM2 is a critical rate-limiting enzyme in the glycolytic pathway (23). Glycolysis not only provides essential energy for tumor cell growth but also creates a favorable microenvironment, with key enzymes playing crucial roles in the process. Intriguingly, research into the role of LINC01410 in glycolysis reveals that it indirectly regulates downstream critical enzymes through a sponge mechanism, positioning LINC01410 as a pivotal player in glycolysis with significant research potential and value. Previous studies have demonstrated that PKM2 is highly expressed in proliferating cells, particularly in tumor cells, where it plays a crucial role in the Warburg effect as well as in tumorigenesis, invasion, and metastasis (46, 47). In research focused on esophageal cancer, experimental findings revealed that exosome-derived LINC01410 acts as a molecular sponge for miR-122-5p, thereby indirectly promoting the upregulation of PKM2 in esophageal cancer cells. This, in turn, facilitates the glycolytic process (as shown in **Figure 4**).

Cancer cells primarily rely on aerobic glycolysis to generate energy required for cellular processes (26). This metabolic pattern leads to markedly increased glucose consumption and lactate production. In studies of neuroblastoma, silencing LINC01410 suppressed glucose consumption and lactate production in GI-LI-N and SK-N-BE cells (26). Similarly, treatment with the glycolysis inhibitor 2-deoxyglucose (2-DG) also reduced glucose consumption and lactate production in these cells (26). Notably, 2-DG treatment abrogated the pro-survival effect of LINC01410, suggesting that LINC01410 knockdown enhances radiosensitivity by inhibiting glycolysis. Further investigations revealed that silencing miR-545-3p partially attenuated the inhibitory effects of LINC01410 knockdown on glucose consumption and lactate production (26).HK2, a key isoform within the hexokinase family, has been reported to be overexpressed, driving the glucose metabolic rate essential for tumor growth in various cancers (48, 49). Taken together LINC01410 may regulate HK2 expression by sponging miR-545-3p, thereby promoting the progression of glycolysis (26). This regulation, in turn, influences neuroblastoma cell proliferation and radiosensitivity.

However, in studies related to DN, the overexpression of LINC01410 indirectly promotes the upregulation of PKM2. As a key glycolytic enzyme, PKM2 is essential for preserving podocyte function and mitigating the progression of DN. Thus, the upregulation of LINC01410 may serve to delay the progression of DN (as shown in **Figure 4**). In summary, LINC01410 indirectly regulates key enzymes involved in glycolysis, thereby promoting tumor cell proliferation. However, in the context of DN, it appears to improve podocyte function. Consequently, the downregulation of LINC01410 expression in tumor cells may effectively hinder the progression of glycolysis and slow tumor cell proliferation, whereas elevated LINC01410 expression can significantly contribute to the improvement of DN.

### 3.4 The role of LINC01410 in LD accumulation and its impact on cancer lymph node metastasis

Cervical cancer (CC) ranks among the top four cancers affecting women worldwide (50). Notably, lymph node metastasis (LNM) is a common occurrence in CC patients, often present even in the early stages of the disease, and remains a leading cause of cancer-related mortality in this population (51). The five-year survival rate for CC patients without LNM is 80-90%, whereas it drops dramatically to 50-65% in those with LNM (52, 53). To improve the prognosis of patients with CC and LNM, there is an urgent need to explore the molecular mechanisms underlying CC and identify effective therapeutic targets (21).

Metabolic reprogramming has been widely recognized as a hallmark of cancer initiation and metastasis (54). Although the Warburg effect has long been regarded as a classical feature of tumor metabolism, increasing evidence indicates that heterogeneous metabolic phenotypes exist across different cancer types and stages of progression (55, 56).In cervical cancer (CC), the abnormal accumulation of lipid droplets (LD) plays a critical role in LNM. LDs function not only as the major intracellular reservoirs of lipids but also as substrates for membrane biosynthesis and as signaling hubs, regulating inflammatory responses and metastasis-associated pathways, thereby exerting a profound impact on the tumor microenvironment (57–60). Among them, fatty acid (FA) metabolic reprogramming is considered to provide cancer cells with a metabolic advantage for invasion and metastasis (61, 62).

In CC, the LINC01410/miR-532-5p/FASN metabolic axis has been identified as a key pathway driving LD dynamics and metabolic reprogramming. Low levels of miR-532-5p are closely associated with higher lymphatic vessel density (D2-40–positive) and the tube formation ability of human lymphatic endothelial cells (HLECs), suggesting that it promotes lymphangiogenesis and metastasis by remodeling the tumor microenvironment (21).Mechanistic studies indicate that miR-532-5p directly targets the 3′UTR of fatty acid synthase (FASN), downregulating its expression and thereby inhibiting lipid synthesis and LD accumulation (21). Overexpression of miR-532-5p not only reduces levels of triglycerides (TAG) and phosphatidylinositol (PL) but also downregulates FASN, ACC1, and PLIN2, while upregulating fatty acid oxidation–related enzymes CPT1A and ACOX1, demonstrating a bidirectional regulatory effect on lipid metabolism (21). In contrast, loss of miR-532-5p leads to substantial LD accumulation, further enhancing cellular invasiveness.

**Figure 5:**
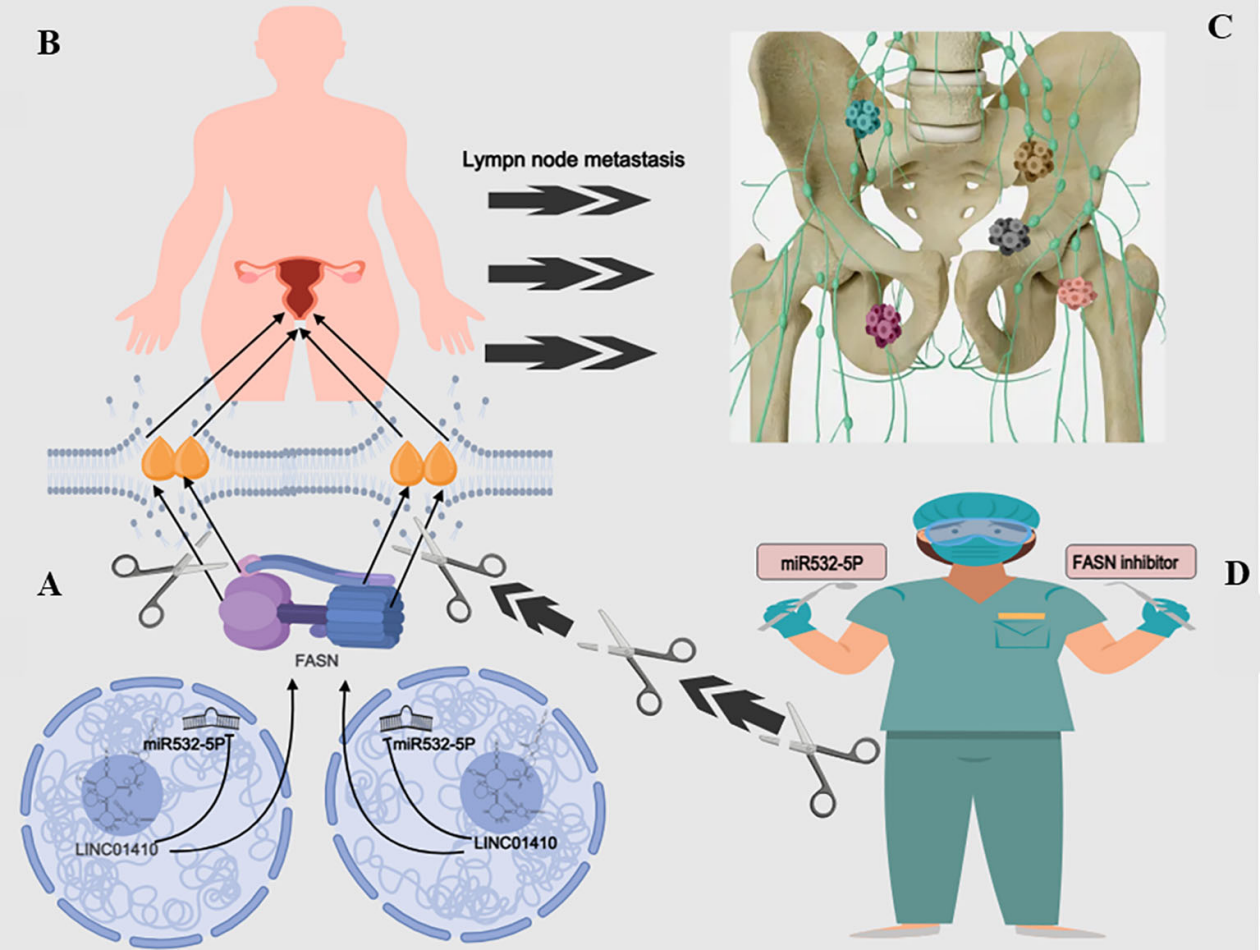
**(A)** illustrates that LINC01410 promotes the expression of fatty acid synthase (FASN) by downregulating miR-532-5p. This regulatory mechanism leads to the accumulation of lipid droplets (LDs) shown in **(B)**, which contributes to the initiation and progression of cervical cancer. Consequently, as depicted in **(C)**, this process facilitates lymph node metastasis (LNM). Interestingly, combined treatment with the fatty acid synthase (FASN) inhibitor Orlistat effectively blocks this oncogenic mechanism, as demonstrated in **(D)**. This figure was created using Medpeer (www.medpeer.cn).

Histological and database analyses further confirm that FASN is highly expressed in CC tissues and in patients with LNM, primarily localizing to the cell membrane and cytoplasm, and positively correlates with the LD marker protein PLIN2 (21). Silencing of PLIN2 significantly inhibits the invasiveness of CC cells, whereas restorative expression of FASN can reverse the lipid metabolic abnormalities induced by miR-532-5p overexpression (21). It is noteworthy that the long non-coding RNA LINC01410 binds to miR-532-5p through a molecular sponge mechanism, thereby relieving its inhibition of FASN, ultimately accelerating LD accumulation and fatty acid metabolic reprogramming, and promoting EMT, invasion, and LNM of tumor cells (21). More importantly, the combined metabolic-targeting strategy demonstrates potential clinical value: the co-treatment of miR-532-5p and the FASN inhibitor Orlistat significantly suppressed tumor growth and LNM *in vivo* (18) (as shown in **Figure 5**).

In summary, the LINC01410/miR-532-5p/FASN metabolic axis drives lymphangiogenesis and cervical cancer metastasis within the tumor microenvironment by regulating LD dynamics and fatty acid metabolic reprogramming (21). This pathway not only reveals a critical link between metabolic reprogramming and metastasis but also provides novel therapeutic strategies for targeting LD accumulation and key metabolic enzymes.

### 3.5 The impact of LINC01410 on protein expression in cancer

In cancer, the aberrant expression of proteins plays a pivotal role in the progression of the disease, influencing key cellular processes such as the cell cycle, apoptosis, proliferation, and invasion. Notably, the dysregulation of cadherins and vimentin significantly enhances the migration of cancer cells and the EMT, thereby facilitating metastatic potential. In studies of bladder cancer, it has been demonstrated that the silencing of LINC 01410 significantly reduces the expression levels of Snail 1, vimentin, and N-cadherin, while the expression of E-cadherin, impaired by LINC01410, is notably restored (51). In osteosarcoma, elevated expression of LINC01410 was found to induce the upregulation of N-cadherin, vimentin, and cyclin D1 in MG-63 cells (16). In cholangiocarcinoma, studies have revealed that the knockout of LINC 01410 results in a significant reduction in the expression levels of SMAD 5. Furthermore, silencing of LINC01410 was found to promote the expression of E-cadherin while inhibiting the expression of N-cadherin (31). In esophageal cancer, overexpression of LINC01410 was associated with upregulation of Snail and vimentin and effectively promoted EMT in TE-1 cells. In the LINC01410 overexpression group, the levels of PKM2, Snail, and vimentin were markedly elevated, while E-cadherin expression was reduced (23). In neuroblastoma, LINC01410 enhances the expression of WEE1 by acting as a sponge for miR-506-3p, thereby promoting the progression of the cell cycle (25). In neuroblastoma, LINC01410 enhances the expression of WEE1 by acting as a sponge for miR-506-3p, thereby promoting the progression of the cell cycle (33). In studies on pancreatic cancer, it was observed that silencing LINC01410 expression resulted in a decrease in IFITM3 protein levels in BxPC-3 cells, along with a downregulation of cell proliferation-associated proteins, including CDK6, Cyclin D2, and proliferating cell nuclear antigen (PCNA). Furthermore, the expression of metastasis-related proteins, such as vimentin and N-cadherin, was also significantly reduced (6). In glioma, studies have shown that the deletion of LINC01410 results in a marked reduction in the expression of Cyclin D1, CDK6, and Bcl-2, while simultaneously elevating the expression of Bax. These findings suggest that the downregulation of LINC01410 disrupts the cell cycle and promotes apoptotic pathways (29). In conclusion, the aberrant expression of cancer-associated proteins significantly influences the migration, invasion, EMT, as well as the cell cycle and apoptosis of cancer cells. Notably, the expression levels of these proteins are modulated by the dysregulated expression of LINC01410. Therefore, both LINC01410 and cancer-related proteins present considerable potential as biomarkers and therapeutic targets in cancer treatment, offering profound clinical significance.

## 4 The associated signaling pathways of LINC01410 in various diseases

**Figure 6:**
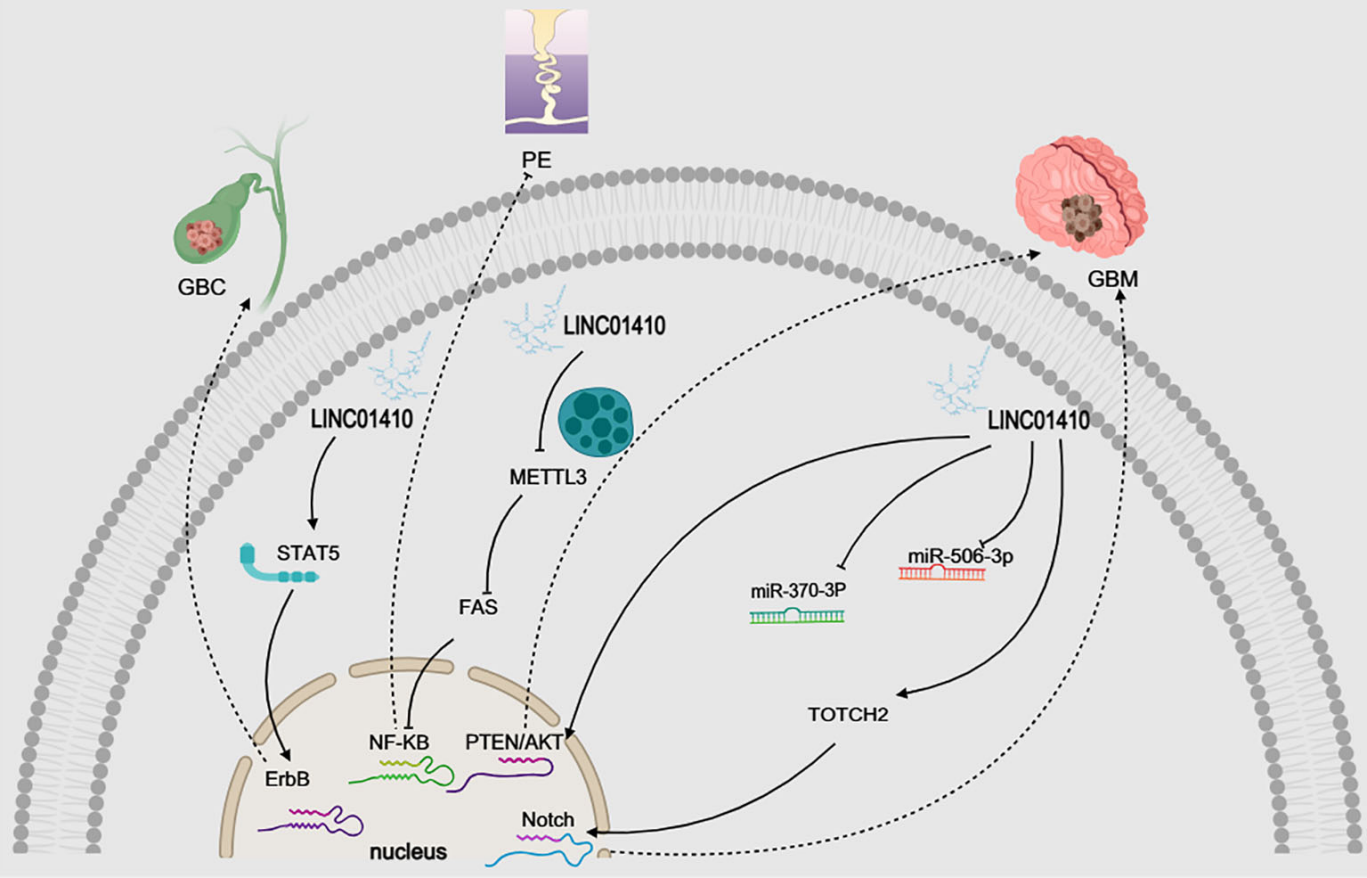
**(A)** illustrates that LINC01410 promotes the expression of fatty acid synthase (FASN) by downregulating miR-532-5p. This regulatory mechanism leads to the accumulation of lipid droplets (LDs) shown in **(B)**, which contributes to the initiation and progression of cervical cancer. Consequently, as depicted in **(C)**, this process facilitates lymph node metastasis (LNM). Interestingly, combined treatment with the fatty acid synthase (FASN) inhibitor Orlistat effectively blocks this oncogenic mechanism, as demonstrated in **(D)**. This figure was created using Medpeer (www.medpeer.cn).

An increasing body of evidence suggests that lncRNAs play a pivotal role in the regulation of various signaling pathways, and research in this field offers new avenues for the development of effective targeted therapies (63). LINC01410 has been shown to regulate four distinct signaling pathways, thereby promoting the initiation and progression of cancer. The signaling pathways associated with LINC 01410 in cancer include the PTEN/AKT pathway, Notch signaling pathway, ErbB signaling pathway, and NF-κB signaling pathway(as shown in **Figure 6**).

### 4.1 NOTCH signaling pathway

NOTCH signaling is involved in a broad range of biological processes across species, including organogenesis, tissue function, and repair. Consequently, aberrant NOTCH signaling can result in pathological outcomes (64). Variants of NOTCH have also been identified in ancient humans, where they are linked to the regulation of brain size (65). In gliomas, Analysis using the GEPIA2 database revealed that LINC01410 expression was significantly higher in tumor tissues compared with normal tissues, and it was also highly expressed in multiple glioma cell lines (SHG44, T98G, LN229, A172) (29).Luciferase reporter assays confirmed the interaction between LINC01410 and miR-506-3p, and silencing LINC01410 markedly reduced the activity of the Notch signaling pathway. Further investigations revealed that NOTCH2, a key molecule in the Notch pathway, is a direct target of miR-506-3p. RT-qPCR and Western blot analyses demonstrated that LINC01410 downregulation led to decreased mRNA and protein levels of NOTCH2 (29).Functional validation experiments demonstrated that the miR-506-3p mimic reduced the luciferase activity of wild-type NOTCH2, whereas no significant change was observed in the mutant construct; moreover, inhibition of miR-506-3p reversed the downregulation of NOTCH2 induced by LINC01410 silencing (29).In summary, LINC01410 competitively binds to miR-506-3p to upregulate NOTCH2 expression, thereby activating the Notch signaling pathway(as shown in **Figure 6**).

### 4.2 PTEN/AKT signaling pathway

The PTEN/AKT pathway has been shown to regulate a variety of cellular functions, including proliferation, migration, and apoptosis, with PTEN exerting its regulatory effects through its target molecule, AKT (66, 67). Furthermore, the PTEN/AKT pathway has been implicated in several cancers, including breast cancer, hepatocellular carcinoma, and chronic myelogenous leukemia (68–70). There is also evidence supporting the involvement of the PTEN/AKT pathway in GBM (71). In GBM, A dual-luciferase reporter assay confirmed the targeting relationship between LINC01410 and microRNA (miR)-370-3p (28). Further investigations revealed that silencing LINC01410 reduced the p-AKT/AKT ratio; however, downregulation of miR-370-3p increased the p-AKT/AKT ratio (28). In addition, suppression of miR-370-3p reversed the effects of LINC01410 silencing on the expression of PTEN/AKT pathway–related factors in TMZ-resistant GBM cells (28). (as shown in **Figure 6**).

### 4.3 ErbB signaling pathway

Approximately three decades ago, ERBB receptors were implicated in the pathogenesis of human cancers (72). Since then, biomedical researchers have gained substantial insight into the biological foundations of cancer dependence on aberrant ERBB receptor signaling (72). In gallbladder cancer, research has demonstrated that LINC01410 is a significant lncRNA that promotes GBC progression through the activation of the ErbB signaling pathway (31). In addition, studies have shown that STAT5 is a multifunctional transcription factor involved in various processes (73, 74). It is also a tumor accelerator in various malignancies, including GBC, as the persistent activation of STAT5 is a major culprit in tumorigenesis (75, 76). Through RNA pull-down assays combined with mass spectrometry, we identified signal transducer and activator of transcription 5 (STAT5) as a LINC01410-associated protein (31). Subsequent Western blot analysis further validated the interaction between LINC01410 and STAT5 (31). Moreover, RNA immunoprecipitation (RIP) assays demonstrated that STAT5 protein was markedly enriched with LINC01410 in NOZ cells, confirming that STAT5 is regulated by LINC01410 and may serve as its downstream target (31).Interestingly, Studies have demonstrated that STAT5 can result in stimulation of ErbB signaling pathway (77, 78). STAT5 is an important transcription factor in ErbB signaling pathway, which is involved in the regulation of cell proliferation, migration, differentiation, apoptosis (79). LINC01410 promoted GBC progression by regulating STAT5 expression and activating ErbB signaling pathway (31)(as shown in **Figure 6**). However, the role of the ErbB signaling pathway in promoting the tumorigenesis and progression mediated by LINC01410 remains unclear, and this issue requires further investigation in future studies.

### 4.4 NF-κB signaling pathway

NF-κB is a transcription factor widely present across various cellular environments, coordinating a critical regulatory spectrum that includes immune modulation, cell proliferation, apoptosis, and inflammation (80). NF-κB is considered a downstream target of FAS/FASL (81). we identified that heightened expression of LINC01410 notably diminished the protein abundance of NF-κB. Additionally, LINC01410 suppressed the expression of the pro-apoptotic gene BAX while augmenting the protein expression of the anti-apoptotic gene BCL-2 (11). In PE, the results suggest that LINC01410 may inhibit trophoblast cell apoptosis by suppressing the gastric L3/FAS/NF-κB pathway (11) (as shown in **Figure 6**). These findings indicate that LINC01410 could be a promising therapeutic strategy for the prevention or mitigation of PE, a condition for which definitive targeted treatments are currently lacking.

## 5 The regulatory role of LINC01410 in non-malignant diseases

### 5.1 Diabetic nephropathy

**Figure 7:**
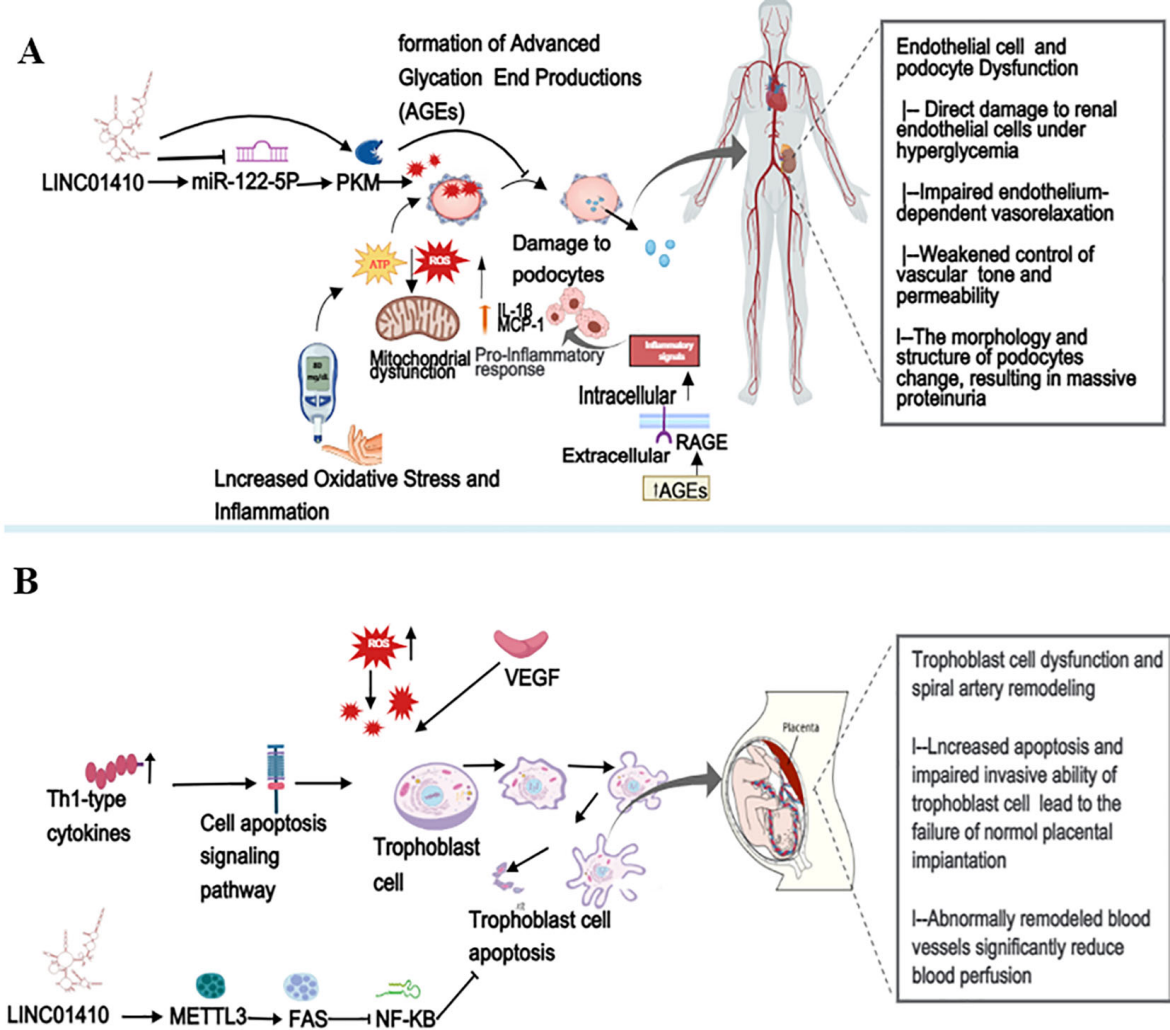
**(A)** illustrates how factors such as dysregulated glucose metabolism and oxidative stress lead to damage and functional abnormalities in renal endothelial cells and podocytes, subsequently resulting in renal tissue injury and the onset of significant proteinuria. However, the upregulation of LINC01410 promotes the increase of its downstream target, PKM, which effectively improves podocyte function and delays the progression of DN. **(B)** shows that factors such as oxidative stress lead to increased apoptosis and inadequate invasion of trophoblast cells, resulting in shallow placental implantation and abnormal vascular remodeling, which in turn promote the onset of PE. Interestingly, the elevated expression of LINC01410 can suppress the inhibitory effects of METTL3 and FAS overexpression on trophoblast cell invasion and the promotion of apoptosis. Furthermore, the inactivation of the NF-κB signaling pathway could effectively prevent the occurrence and progression of PE. This figure was created using Medpeer (www.medpeer.cn).

Diabetic nephropathy (DN) is a major microvascular complication of diabetes, occurring in 20% to 40% of diabetic patients, and is the most common cause of end-stage renal failure (82). Therefore, identifying new diagnostic biomarkers and therapeutic targets for DN is of paramount importance. Based on bioinformatics analysis, LINC01410, MAFB, and FOSL1 were selected. Interestingly, as the disease progresses, the expression of LINC01410 significantly decreases, while the expression of FOSL1 and MAFB notably increases (30). Given the significant changes in the expression of LINC01410, FOSL1, and MAFB in DN patients compared to those with normal albuminuria, it can be suggested that these RNAs are involved in the early stages of the disease (30). In addition, studies show that elevated LINC01410 leads to an increase in PKM2 levels through the LINC01410/miR-122-5p/PKM2 axis (23). It is worth noting that PKM2, as a glycolytic enzyme, is essential for improving podocyte function and delaying the progression of DN (83). Therefore, LINC01410 can indirectly promote the elevation of PKM2 levels, thereby improving podocyte function and delaying the progression of DN (as shown in **Figure 7**). The aberrant expression of LINC01410 may be linked to the pathogenesis of the disease, and it has been found that increasing LINC01410 expression could potentially prevent the progression of the disease. LINC01410 may be regarded as a promising diagnostic and prognostic biomarker, as well as a potential therapeutic target for DN.

### 5.2 Preeclampsia

The current medical field is clearly lacking effective treatments or preventive interventions for preeclampsia (PE), primarily due to the mysterious nature of its pathogenesis (84). Therefore, studying its complex pathogenesis and identifying effective therapeutic targets is of paramount importance. Current research has shown that the distinctive physiological characteristics of PE include increased trophoblast cell apoptosis and reduced trophoblast invasion, ultimately leading to suboptimal remodeling of the spiral arteries (85). Therefore, genes that regulate trophoblast cell behavior play a crucial role in the progression and pathogenesis of PE (86). Studies have found that the high expression of LINC01410 significantly enhances the invasive capacity of HTR-8/Svneo cells while reducing their apoptosis rate, leading to a marked decrease in L3 mRNA levels (11). Increased expression levels of FAS promote cell apoptosis, reduce cell viability, and impair the migratory ability of human trophoblast cells (87). N6-methyladenosine (m6A) is a prevalent and dynamic RNA modification that exerts significant influence on a variety of biological processes and diseases (88). Moreover, the interplay between m6A and ncRNAs significantly contributes to the regulatory mechanisms governing disease progression (89). In summary, a strong interrelationship exists among LINC 01410, METTL3, m6A, and FAS, all of which play pivotal roles in the development of PE. The high expression of LINC 01410 can counteract the inhibitory effects of METTL3 and FAS overexpression on trophoblast cell invasion and mitigate the promotion of cell apoptosis. Intriguingly, METTL3 is regarded as a pioneering and essential m6A RNA methyltransferase, orchestrating the addition of m6A modifications to RNA molecules (90). Therefore, LINC01410 may represent a promising therapeutic target for the prevention or alleviation of PE. In conclusion, upregulating LINC 01410 to inhibit the expression of METTL3 and FAS, while concurrently inactivating the NF-κB signaling pathway, could serve as an effective strategy to prevent the onset and progression of PE(as shown in **Figure 7**).

## 6 Conclusion and future perspectives

### 6.1 Current Challenges and the Emerging Role of LINC01410

While traditional therapeutic approaches have witnessed significant advancements, the clinical treatment of cancer still faces a multitude of challenges. High recurrence rates and suboptimal treatment precision remain substantial barriers to improving the prognosis of cancer patients. In light of this, LINC01410 emerges as a promising biomarker for the early prediction of cancer onset, as well as a novel and potentially transformative therapeutic target. However, research on LINC01410 remains in its nascent stages.

### 6.2 Mechanistic insights and experimental limitations

Notably, we have observed that elevated expression of LINC01410 consistently accelerates the progression of cancer yet simultaneously appears to facilitate improvements in conditions such as preeclampsia and diabetic nephropathy. This paradoxical behavior suggests that LINC01410 may exert opposing clinical effects in malignant versus benign diseases. However, current investigations are limited to a relatively narrow range of conditions, and several of its underlying mechanisms remain poorly understood. Further research is therefore required to refine our understanding, particularly regarding the detailed mechanisms by which LINC01410 silencing enhances apoptosis in TMZ-resistant GBM cells. In the field of gallbladder cancer research, the precise mechanisms underlying the overexpression of LINC01410 in GBC remain poorly understood, and the exact nature of its interaction with STAT5 requires further clarification. Moreover, many studies have been confined to *in vitro* experiments, and whether the observed mechanisms hold true *in vivo* remains to be validated in future investigations. Additionally, the upstream molecular pathways that regulate LINC01410 expression are yet to be fully characterized.

### 6.3 Translational challenges and future therapeutic strategies

While LINC01410 presents a promising candidate as a therapeutic target in cancer treatment, its clinical efficacy has yet to be substantiated, and no targeted therapies aimed at LINC01410 have been developed or employed thus far—issues that necessitate further exploration. Furthermore, the potential associations between LINC01410 and drug sensitivity, as well as responsiveness to radiation therapy, remain largely unexplored, offering substantial avenues for future research.

Another limitation lies in the homogeneity of experimental samples; for example, osteosarcoma studies have been based on only 50 OS tissue samples, bladder cancer investigations have involved a sample size of just 60 cases, and colon cancer research has primarily utilized a small cohort of serum samples from a single institution. These constraints may impact the accuracy and reliability of the findings, highlighting the need for more diverse and rigorous study designs.

### 6.4 Outlook: overcoming translational barriers

Given the growing evidence supporting the oncogenic role of LINC01410, strategies aimed at its targeted inhibition have attracted increasing attention. At the technical level, several approaches have demonstrated feasibility, including antisense oligonucleotides (ASOs), RNA interference (RNAi), and CRISPR-Cas genome editing systems (91, 92). Nevertheless, translating these technologies into effective LINC01410-targeted therapies remains challenging, particularly with respect to achieving efficient *in vivo* delivery, minimizing off-target effects, and ensuring long-term safety and biocompatibility (93). In recent years, nanoparticle- and lipid-based delivery systems, along with tissue-specific modifications, have shown promise in partially overcoming these obstacles (94). Looking forward, it will be essential to rigorously evaluate the safety and therapeutic efficacy of LINC01410-directed interventions in preclinical animal models and to advance these findings toward clinical translation. Thus, although LINC01410 represents a promising therapeutic target with substantial potential, addressing the translational bottlenecks remains a critical step toward its successful implementation in clinical oncology.

## Glossary

OS: osteosarcoma
TC: thyroid cancer
EC: endometrial cancer
GBM: glioblastoma
NB: neuroblastoma
GBC: gallbladder cancer
DN: Diabetic nephropathy
ceRNAs: competitive endogenous RNAs
EMT: epithelial-to-mesenchymal transition
CAFs: cancer-associated fibroblasts
LD: lipid droplets
FA: fatty acid
STAT5: Signal Transducer and Activator of Transcription 5
M6A: N6-methyladenosine
SMAD: SMA-Mothers against decapentaplegic
CHD7: Chromodomain Helicase DNA Binding Protein 7
HK2: Hexokinase 2
HMGA2: High Mobility Group AT-hook 2
VOPP1: Viral Oncogene Overexpressed Protein 1
AGEs: Advanced Glycation End-products
MAFB: Advanced Glycation End-products
MAFB: MAF BZIP Transcription Factor B
BC: bladder cancer
CC: cervical cancer
ESCC: esophageal squamous cell carcinoma
CCA: cholangiocarcinoma
CRC: colorectal cancer
NSCLC: Non-Small Cell Lung Cancer
PE: Preeclampsia
ncRNAs: Non-coding RNAs
TMZ: temozolomide
LNM: lymph node metastasis
FASN: fatty acid synthase
AUC: Area Under the Curve
METTL3: Methyltransferase Like 3
FASL: Fas Ligand
FOXM1: Forkhead box M1
PKM2: Pyruvate Kinase M2
NDRG3: N-myc Downstream-Regulated Gene 3
WEE1: WEE1 G2 Checkpoint Kinase
Snail1: SNAIL Family Transcriptional Repressor 1
VEGF: Vascular Endothelial Growth Factor
PCNA: cell nuclear antigen

## References

[1] Essa H Dobson R Wright D Lip GYH . Hypertension management in cardio-oncology. J Hum Hypertens. (2020) 34:673–81. doi: 10.1038/s41371-020-0391-8, PMID: 32747676 PMC7398285

[2] Ferlay J Colombet M Soerjomataram I Dyba T Randi G Bettio M . Cancer incidence and mortality patterns in europe: estimates for 40 countries and 25 major cancers in 2018. Eur J Cancer. (2018) 103:356–87. doi: 10.1016/j.ejca.2018.07.005, PMID: 30100160

[3] Fidler MM Soerjomataram I Bray F . A global view on cancer incidence and national levels of the human development index. Int J Cancer. (2016) 139:2436–46. doi: 10.1002/ijc.30382, PMID: 27522007

[4] Torre LA Siegel RL Ward EM Jemal A . Global cancer incidence and mortality rates and trends–an update. Cancer Epidemiol Biomarkers Prev. (2016) 25:16–27. doi: 10.1158/1055-9965.Epi-15-0578, PMID: 26667886

[5] Jathar S Kumar V Srivastava J Tripathi V . Technological developments in lncrna biology. Adv Exp Med Biol. (2017) 1008:283–323. doi: 10.1007/978-981-10-5203-3_10, PMID: 28815544

[6] Cai M Xu L Shen L Zhang J . the expression of long non-coding rna-linc01410 in pancreatic cancer and its effect on proliferation and migration of pancreatic cancer cells. Zhonghua Yi Xue Za Zhi. (2019) 99:1406–11. doi: 10.3760/cma.j.issn.0376-2491.2019.18.010, PMID: 31137129

[7] Anastasiadou E Jacob LS Slack FJ . Non-coding rna networks in cancer. Nat Rev Cancer. (2018) 18:5–18. doi: 10.1038/nrc.2017.99, PMID: 29170536 PMC6337726

[8] Jiang X Ma N Wang D Li F He R Li D . Metformin inhibits tumor growth by regulating multiple mirnas in human cholangiocarcinoma. Oncotarget. (2015) 6:3178–94. doi: 10.18632/oncotarget.3063, PMID: 25605008 PMC4413646

[9] Slack FJ Chinnaiyan AM . The role of non-coding rnas in oncology. Cell. (2019) 179:1033–55. doi: 10.1016/j.cell.2019.10.017, PMID: 31730848 PMC7347159

[10] Cong Z Diao Y Xu Y Li X Jiang Z Shao C . Long non-coding rna linc00665 promotes lung adenocarcinoma progression and functions as cerna to regulate akr1b10-erk signaling by sponging mir-98. Cell Death Dis. (2019) 10:84. doi: 10.1038/s41419-019-1361-3, PMID: 30692511 PMC6349882

[11] Yang Y Chen M Lan R Gong H . Linc01410 accelerates the invasion of trophoblast cells by modulating mettl3/fas. Mol Biol Rep. (2024) 51:895. doi: 10.1007/s11033-024-09834-6, PMID: 39115693 PMC11310249

[12] Birgani MT Hajjari M Shahrisa A Khoshnevisan A Shoja Z Motahari P . Long non-coding rna snhg6 as a potential biomarker for hepatocellular carcinoma. Pathol Oncol Res. (2018) 24:329–37. doi: 10.1007/s12253-017-0241-3, PMID: 28508329

[13] Jia B Qiu X Chen J Sun X Zheng X Zhao J . A feed-forward regulatory network lncpcat1/mir-106a-5p/E2f5 regulates the osteogenic differentiation of periodontal ligament stem cells. J Cell Physiol. (2019) 234:19523–38. doi: 10.1002/jcp.28550, PMID: 30997692 PMC6767496

[14] Shao S Wang C Wang S Zhang H Zhang Y . Lncrna stxbp5-as1 suppressed cervical cancer progression via targeting mir-96-5p/pten axis. BioMed Pharmacother. (2019) 117:109082. doi: 10.1016/j.biopha.2019.109082, PMID: 31212131

[15] Ma W Zhao X Xue N Gao Y Xu Q . The linc01410/mir-122-5p/ndrg3 axis is involved in the proliferation and migration of osteosarcoma cells. IUBMB Life. (2021) 73:705–17. doi: 10.1002/iub.2452, PMID: 33583123

[16] Xu Q He L Ma L Fan L Yan L Zhao X . Linc01410 accelerated the invasion and proliferation of osteosarcoma by sponging mir-3128. Aging (Albany NY). (2020) 12:24957–66. doi: 10.18632/aging.103464, PMID: 33401246 PMC7803582

[17] Ma W Gao Y Zhang J Yao X Jia L Xu Q . Long noncoding rna linc01410 promotes tumorigenesis of osteosarcoma cells via mir-497-5p/hmga2 axis. J Biochem Mol Toxicol. (2021) 35:e22921. doi: 10.1002/jbt.22921, PMID: 34605103

[18] Guo W Gai Q Ma Y Shan Z Wu J . Linc01410 leads the migration, invasion and emt of bladder cancer cells by modulating mir-4319/snail1. Cancer Cell Int. (2021) 21:429. doi: 10.1186/s12935-021-02119-z, PMID: 34391433 PMC8364693

[19] Wang G Wang X Jin Y . Linc01410/mir-3619-5p/foxm1 feedback loop regulates papillary thyroid carcinoma cell proliferation and apoptosis. Cancer Biother Radiopharm. (2019) 34:572–80. doi: 10.1089/cbr.2019.2854, PMID: 31644316

[20] Liu F Wen C . Linc01410 knockdown suppresses cervical cancer growth and invasion via targeting mir-2467-3p/vopp1 axis. Cancer Manag Res. (2020) 12:855–61. doi: 10.2147/cmar.S236832, PMID: 32104067 PMC7008191

[21] Shang C Li Y He T Liao Y Du Q Wang P . The prognostic mir-532-5p-correlated cerna-mediated lipid droplet accumulation drives nodal metastasis of cervical cancer. J Adv Res. (2022) 37:169–84. doi: 10.1016/j.jare.2021.09.009, PMID: 35499057 PMC9040090

[22] Lu M Ding N Zhuang S Li Y . Linc01410/mir-23c/chd7 functions as a cerna network to affect the prognosis of patients with endometrial cancer and strengthen the Malignant properties of endometrial cancer cells. Mol Cell Biochem. (2020) 469:9–19. doi: 10.1007/s11010-020-03723-9, PMID: 32314193

[23] Shi Z Jiang T Cao B Sun X Liu J . Caf-derived exosomes deliver linc01410 to promote epithelial-mesenchymal transition of esophageal squamous cell carcinoma. Exp Cell Res. (2022) 412:113033. doi: 10.1016/j.yexcr.2022.113033, PMID: 35041823

[24] Jiang T Wang C Zhu Y Han H . Linc01410 promotes cell proliferation and migration of cholangiocarcinoma through modulating mir-124-3p/smad5 axis. J Gene Med. (2020) 22:e3162. doi: 10.1002/jgm.3162, PMID: 31951299

[25] Mi J Han Y Zhang J Hao X Xing M Shang C . Long noncoding rna linc01410 promotes the tumorigenesis of neuroblastoma cells by sponging microrna-506-3p and modulating wee1. Cancer Med. (2020) 9:8133–43. doi: 10.1002/cam4.3398, PMID: 32886453 PMC7643657

[26] Mou L Wang L Zhang S Wang Q . Long noncoding rna linc01410 suppresses tumorigenesis and enhances radiosensitivity in neuroblastoma cells through regulating mir-545-3p/hk2 axis. Onco Targets Ther. (2021) 14:3225–38. doi: 10.2147/ott.S297969, PMID: 34040388 PMC8140916

[27] Xu J Wang L Wang Q . High expression of long noncoding rna 01410 serves as a potential diagnostic and prognostic marker in patients with colorectal cancer. Clin Lab. (2021) 67. doi: 10.7754/Clin.Lab.2020.200805, PMID: 33978387

[28] Fu T Yang Y Mu Z Sun R Li X Dong J . Silencing lncrna linc01410 suppresses cell viability yet promotes apoptosis and sensitivity to temozolomide in glioblastoma cells by inactivating pten/akt pathway via targeting mir-370-3p. Immunopharmacol Immunotoxicol. (2021) 43:680–92. doi: 10.1080/08923973.2021.1966031, PMID: 34435542

[29] Zhao X Shen F Yang B . Lncrna linc01410 induced by myc accelerates glioma progression via sponging mir-506-3p and modulating notch2 expression to motivate notch signaling pathway. Cell Mol Neurobiol. (2022) 42:1513–21. doi: 10.1007/s10571-021-01042-1, PMID: 33712887 PMC11421750

[30] Asadollahi S Hadizadeh M Namiranian N Kalantar SM Firoozabadi AD Injinari N . Misexpression of linc01410, fosl1, and mafb in peripheral blood mononuclear cells associated with diabetic nephropathy. Gene. (2023) 862:147265. doi: 10.1016/j.gene.2023.147265, PMID: 36764337

[31] Lu L Zhang S Song Z Lu W Wang Z Zhou Y . Long non-coding rna linc01410 promoted tumor progression via the erbb signaling pathway by targeting stat5 in gallbladder cancer. Front Oncol. (2021) 11:659123. doi: 10.3389/fonc.2021.659123, PMID: 34322379 PMC8312242

[32] Luo J Guo Y Liu X Yang X Xiao F Zhou M . Long non-coding rna linc01410 promotes colon cancer cell proliferation and invasion by inhibiting mir-3128. Exp Ther Med. (2018) 16:4824–30. doi: 10.3892/etm.2018.6806, PMID: 30546401 PMC6256842

[33] Saleh AA Elghobashy YA Kasemy ZA Hegazy A AA AL . Impact of dysregulated linc01559 and linc01410 expression on the diagnosis and survival of non-small cell lung cancer. Biochem Genet. (2024) 62:4011–26. doi: 10.1007/s10528-023-10632-1, PMID: 38265621

[34] Zuo W Zhou K Deng M Lin Q Yin Q Zhang C . Linc00963 facilitates acute myeloid leukemia development by modulating mir-608/mmp-15. Aging (Albany NY). (2020) 12:18970–81. doi: 10.18632/aging.103252, PMID: 33012724 PMC7732318

[35] Saw PE Xu X Chen J Song EW . Non-coding rnas: the new central dogma of cancer biology. Sci China Life Sci. (2021) 64:22–50. doi: 10.1007/s11427-020-1700-9, PMID: 32930921

[36] Salmena L Poliseno L Tay Y Kats L Pandolfi PP . A cerna hypothesis: the rosetta stone of a hidden rna language? Cell. (2011) 146:353–8. doi: 10.1016/j.cell.2011.07.014, PMID: 21802130 PMC3235919

[37] Tay Y Rinn J Pandolfi PP . The multilayered complexity of cerna crosstalk and competition. Nature. (2014) 505:344–52. doi: 10.1038/nature12986, PMID: 24429633 PMC4113481

[38] Fujisawa M Moh-Moh-Aung A Zeng Z Yoshimura T Wani Y Matsukawa A . Ovarian stromal cells as a source of cancer-associated fibroblasts in human epithelial ovarian cancer: A histopathological study. PloS One. (2018) 13:e0205494. doi: 10.1371/journal.pone.0205494, PMID: 30304016 PMC6179287

[39] Kalluri R . The biology and function of exosomes in cancer. J Clin Invest. (2016) 126:1208–15. doi: 10.1172/jci81135, PMID: 27035812 PMC4811149

[40] Mathieu M Martin-Jaular L Lavieu G Théry C . Specificities of secretion and uptake of exosomes and other extracellular vesicles for cell-to-cell communication. Nat Cell Biol. (2019) 21:9–17. doi: 10.1038/s41556-018-0250-9, PMID: 30602770

[41] Yang H Fu H Xu W Zhang X . Exosomal non-coding rnas: A promising cancer biomarker. Clin Chem Lab Med. (2016) 54:1871–9. doi: 10.1515/cclm-2016-0029, PMID: 27166723

[42] Li Q Shao Y Zhang X Zheng T Miao M Qin L . Plasma long noncoding rna protected by exosomes as a potential stable biomarker for gastric cancer. Tumour Biol. (2015) 36:2007–12. doi: 10.1007/s13277-014-2807-y, PMID: 25391424

[43] Li Y Zhao Z Liu W Li X . Snhg3 functions as mirna sponge to promote breast cancer cells growth through the metabolic reprogramming. Appl Biochem Biotechnol. (2020) 191:1084–99. doi: 10.1007/s12010-020-03244-7, PMID: 31956955 PMC7320061

[44] Ren J Ding L Zhang D Shi G Xu Q Shen S . Carcinoma-associated fibroblasts promote the stemness and chemoresistance of colorectal cancer by transferring exosomal lncrna H19. Theranostics. (2018) 8:3932–48. doi: 10.7150/thno.25541, PMID: 30083271 PMC6071523

[45] Burk D SChade AL . On respiratory impairment in cancer cells. Science. (1956) 124:270–2. doi: 10.1126/science.124.3215.270, PMID: 13351640

[46] Ma B Chen J Mu Y Xue B Zhao A Wang D . Proteomic analysis of rat serum revealed the effects of chronic sleep deprivation on metabolic, cardiovascular and nervous system. PloS One. (2018) 13:e0199237. doi: 10.1371/journal.pone.0199237, PMID: 30235220 PMC6147403

[47] Wang Y Hao F Nan Y Qu L Na W Jia C . Pkm2 inhibitor shikonin overcomes the cisplatin resistance in bladder cancer by inducing necroptosis. Int J Biol Sci. (2018) 14:1883–91. doi: 10.7150/ijbs.27854, PMID: 30443191 PMC6231221

[48] He HC Bi XC Zheng ZW Dai QS Han ZD Liang YX . Real-time quantitative rt-pcr assessment of pim-1 and hk2 mrna expression in benign prostate hyperplasia and prostate cancer. Med Oncol. (2009) 26:303–8. doi: 10.1007/s12032-008-9120-9, PMID: 19003546

[49] Mathupala SP Ko YH Pedersen PL . Hexokinase ii: cancer’s double-edged sword acting as both facilitator and gatekeeper of Malignancy when bound to mitochondria. Oncogene. (2006) 25:4777–86. doi: 10.1038/sj.onc.1209603, PMID: 16892090 PMC3385868

[50] Sung H Ferlay J Siegel RL Laversanne M Soerjomataram I Jemal A . Global cancer statistics 2020: globocan estimates of incidence and mortality worldwide for 36 cancers in 185 countries. CA Cancer J Clin. (2021) 71:209–49. doi: 10.3322/caac.21660, PMID: 33538338

[51] Wright JD Matsuo K Huang Y Tergas AI Hou JY Khoury-Collado F . Prognostic performance of the 2018 international federation of gynecology and obstetrics cervical cancer staging guidelines. Obstet Gynecol. (2019) 134:49–57. doi: 10.1097/aog.0000000000003311, PMID: 31188324 PMC7641496

[52] Chen Y Zhang L Tian J Fu X Ren X Hao Q . Significance of the absolute number and ratio of metastatic lymph nodes in predicting postoperative survival for the international federation of gynecology and obstetrics stage ia2 to iia cervical cancer. Int J Gynecol Cancer. (2013) 23:157–63. doi: 10.1097/IGC.0b013e3182778bcf, PMID: 23221732

[53] Noordhuis MG Fehrmann RS Wisman GB Nijhuis ER van Zanden JJ Moerland PD . Involvement of the tgf-beta and beta-catenin pathways in pelvic lymph node metastasis in early-stage cervical cancer. Clin Cancer Res. (2011) 17:1317–30. doi: 10.1158/1078-0432.Ccr-10-2320, PMID: 21385933

[54] Pavlova NN Thompson CB . The emerging hallmarks of cancer metabolism. Cell Metab. (2016) 23:27–47. doi: 10.1016/j.cmet.2015.12.006, PMID: 26771115 PMC4715268

[55] Nath A Chan C . Genetic alterations in fatty acid transport and metabolism genes are associated with metastatic progression and poor prognosis of human cancers. Sci Rep. (2016) 6:18669. doi: 10.1038/srep18669, PMID: 26725848 PMC4698658

[56] Pascual G Avgustinova A Mejetta S Martín M Castellanos A Attolini CS . Targeting metastasis-initiating cells through the fatty acid receptor cd36. Nature. (2017) 541:41–5. doi: 10.1038/nature20791, PMID: 27974793

[57] Cruz ALS Barreto EA Fazolini NPB Viola JPB Bozza PT . Lipid droplets: platforms with multiple functions in cancer hallmarks. Cell Death Dis. (2020) 11:105. doi: 10.1038/s41419-020-2297-3, PMID: 32029741 PMC7005265

[58] Martin S Parton RG . Lipid droplets: A unified view of a dynamic organelle. Nat Rev Mol Cell Biol. (2006) 7:373–8. doi: 10.1038/nrm1912, PMID: 16550215

[59] Thiam AR Farese RV Jr. Walther TC . The biophysics and cell biology of lipid droplets. Nat Rev Mol Cell Biol. (2013) 14:775–86. doi: 10.1038/nrm3699, PMID: 24220094 PMC4526153

[60] Wright HJ Hou J Xu B Cortez M Potma EO Tromberg BJ . Cdcp1 drives triple-negative breast cancer metastasis through reduction of lipid-droplet abundance and stimulation of fatty acid oxidation. Proc Natl Acad Sci U.S.A. (2017) 114:E6556–e65. doi: 10.1073/pnas.1703791114, PMID: 28739932 PMC5559020

[61] Shang C Wang W Liao Y Chen Y Liu T Du Q . Lnmicc promotes nodal metastasis of cervical cancer by reprogramming fatty acid metabolism. Cancer Res. (2018) 78:877–90. doi: 10.1158/0008-5472.Can-17-2356, PMID: 29229603

[62] Zhang C Liao Y Liu P Du Q Liang Y Ooi S . Fabp5 promotes lymph node metastasis in cervical cancer by reprogramming fatty acid metabolism. Theranostics. (2020) 10:6561–80. doi: 10.7150/thno.44868, PMID: 32550890 PMC7295046

[63] Mirzaei S Zarrabi A Hashemi F Zabolian A Saleki H Ranjbar A . Regulation of nuclear factor-kappab (Nf-Kb) signaling pathway by non-coding rnas in cancer: inhibiting or promoting carcinogenesis? Cancer Lett. (2021) 509:63–80. doi: 10.1016/j.canlet.2021.03.025, PMID: 33838282

[64] Zhou B Lin W Long Y Yang Y Zhang H Wu K . Notch signaling pathway: architecture, disease, and therapeutics. Signal Transduct Target Ther. (2022) 7:95. doi: 10.1038/s41392-022-00934-y, PMID: 35332121 PMC8948217

[65] Lodewijk GA Fernandes DP Vretzakis I Savage JE Jacobs FMJ . Evolution of human brain size-associated notch2nl genes proceeds toward reduced protein levels. Mol Biol Evol. (2020) 37:2531–48. doi: 10.1093/molbev/msaa104, PMID: 32330268 PMC7475042

[66] Li X Dai Y Xu J . Mir-21 promotes pterygium cell proliferation through the pten/akt pathway. Mol Vis. (2018) 24:485–94., PMID: 30967746 PMC6416795

[67] Wang Y Romigh T He X Orloff MS Silverman RH Heston WD . Resveratrol regulates the pten/akt pathway through androgen receptor-dependent and -independent mechanisms in prostate cancer cell lines. Hum Mol Genet. (2010) 19:4319–29. doi: 10.1093/hmg/ddq354, PMID: 20729295 PMC2957324

[68] Jiang XM Yu XN Liu TT Zhu HR Shi X Bilegsaikhan E . Microrna-19a-3p promotes tumor metastasis and chemoresistance through the pten/akt pathway in hepatocellular carcinoma. BioMed Pharmacother. (2018) 105:1147–54. doi: 10.1016/j.biopha.2018.06.097, PMID: 30021351

[69] Nie ZY Yang L Liu XJ Yang Z Yang GS Zhou J . Morin inhibits proliferation and induces apoptosis by modulating the mir-188-5p/pten/akt regulatory pathway in cml cells. Mol Cancer Ther. (2019) 18:2296–307. doi: 10.1158/1535-7163.Mct-19-0051, PMID: 31515296

[70] Wang X Li Y Fan Y Yu X Mao X Jin F . Ptbp1 promotes the growth of breast cancer cells through the pten/akt pathway and autophagy. J Cell Physiol. (2018) 233:8930–9. doi: 10.1002/jcp.26823, PMID: 29856478 PMC6175200

[71] Yang F Zhang C Xu C Fu F Han D Li H . Microrna-559 plays an inhibitory role in the Malignant progression of glioblastoma cells by directly targeting metadherin. Onco Targets Ther. (2019) 12:4415–26. doi: 10.2147/ott.S202309, PMID: 31239710 PMC6556469

[72] Arteaga CL Engelman JA . Erbb receptors: from oncogene discovery to basic science to mechanism-based cancer therapeutics. Cancer Cell. (2014) 25:282–303. doi: 10.1016/j.ccr.2014.02.025, PMID: 24651011 PMC4018830

[73] Huang IH Chung WH Wu PC Chen CB . Jak-stat signaling pathway in the pathogenesis of atopic dermatitis: an updated review. Front Immunol. (2022) 13:1068260. doi: 10.3389/fimmu.2022.1068260, PMID: 36569854 PMC9773077

[74] Pencik J Pham HT Schmoellerl J Javaheri T Schlederer M Culig Z . Jak-stat signaling in cancer: from cytokines to non-coding genome. Cytokine. (2016) 87:26–36. doi: 10.1016/j.cyto.2016.06.017, PMID: 27349799 PMC6059362

[75] Bowman T Garcia R Turkson J Jove R . Stats in oncogenesis. Oncogene. (2000) 19:2474–88. doi: 10.1038/sj.onc.1203527, PMID: 10851046

[76] Turkson J Jove R . Stat proteins: novel molecular targets for cancer drug discovery. Oncogene. (2000) 19:6613–26. doi: 10.1038/sj.onc.1204086, PMID: 11426647

[77] Olayioye MA Neve RM Lane HA Hynes NE . The erbb signaling network: receptor heterodimerization in development and cancer. EMBO J. (2000) 19:3159–67. doi: 10.1093/emboj/19.13.3159, PMID: 10880430 PMC313958

[78] Schulze WX Deng L Mann M . Phosphotyrosine interactome of the erbb-receptor kinase family. Mol Syst Biol. (2005) 1:2005. doi: 10.1038/msb4100012, PMID: 16729043 PMC1681463

[79] Kebenko M Drenckhan A Gros SJ Jücker M Grabinski N Ewald F . Erbb2 signaling activates the hedgehog pathway via pi3k-akt in human esophageal adenocarcinoma: identification of novel targets for concerted therapy concepts. Cell Signal. (2015) 27:373–81. doi: 10.1016/j.cellsig.2014.11.022, PMID: 25435423

[80] Khan D Cornelius JF Muhammad S . The role of nf-Kb in intracranial aneurysm pathogenesis: A systematic review. Int J Mol Sci. (2023) 24:14218. doi: 10.3390/ijms241814218, PMID: 37762520 PMC10531594

[81] Tan S Liu X Chen L Wu X Tao L Pan X . Fas/fasl mediates nf-Kbp65/puma-modulated hepatocytes apoptosis via autophagy to drive liver fibrosis. Cell Death Dis. (2021) 12:474. doi: 10.1038/s41419-021-03749-x, PMID: 33980818 PMC8115181

[82] Hussain S Habib A Hussain MS Najmi AK . Potential biomarkers for early detection of diabetic kidney disease. Diabetes Res Clin Pract. (2020) 161:108082. doi: 10.1016/j.diabres.2020.108082, PMID: 32057966

[83] Qi W Keenan HA Li Q Ishikado A Kannt A Sadowski T . Pyruvate Kinase M2 Activation May Protect against the Progression of Diabetic Glomerular Pathology and Mitochondrial Dysfunction. Nat Med. (2017) 23:753–62. doi: 10.1038/nm.4328, PMID: 28436957 PMC5575773

[84] Liu YH Zhang YS Chen JY Wang ZJ Liu YX Li JQ . Comparative effectiveness of prophylactic strategies for preeclampsia: A network meta-analysis of randomized controlled trials. Am J Obstet Gynecol. (2023) 228:535–46. doi: 10.1016/j.ajog.2022.10.014, PMID: 36283479

[85] Miller EC Wilczek A Bello NA Tom S Wapner R Suh Y . Pregnancy, preeclampsia and maternal aging: from epidemiology to functional genomics. Ageing Res Rev. (2022) 73:101535. doi: 10.1016/j.arr.2021.101535, PMID: 34871806 PMC8827396

[86] Jie Q Chen L Liang J Yang X Sun F Ma Y . Downregulated etv4 inhibits the proliferation, migration, and invasion of trophoblast cells in preeclampsia. Reproduction. (2023) 165:373–81. doi: 10.1530/rep-22-0184, PMID: 36716247

[87] Lan R Yang Y Song J Wang L Gong H . Fas regulates the apoptosis and migration of trophoblast cells by targeting nf-Kb. Exp Ther Med. (2021) 22:1055. doi: 10.3892/etm.2021.10489, PMID: 34434269 PMC8353647

[88] Sendinc E Shi Y . Rna M6a methylation across the transcriptome. Mol Cell. (2023) 83:428–41. doi: 10.1016/j.molcel.2023.01.006, PMID: 36736310

[89] Jin Y Fan Z . New insights into the interaction between M6a modification and lncrna in cancer drug resistance. Cell Prolif. (2024) 57:e13578. doi: 10.1111/cpr.13578, PMID: 37961996 PMC10984110

[90] Zeng C Huang W Li Y Weng H . Roles of mettl3 in cancer: mechanisms and therapeutic targeting. J Hematol Oncol. (2020) 13:117. doi: 10.1186/s13045-020-00951-w, PMID: 32854717 PMC7457244

[91] Crooke ST Liang XH Baker BF Crooke RM . Antisense technology: A review. J Biol Chem. (2021) 296:100416. doi: 10.1016/j.jbc.2021.100416, PMID: 33600796 PMC8005817

[92] Doudna JA Charpentier E . Genome editing. The new frontier of genome engineering with crispr-cas9. Science. (2014) 346:1258096. doi: 10.1126/science.1258096, PMID: 25430774

[93] Ewaisha R Anderson KS . Immunogenicity of crispr therapeutics-critical considerations for clinical translation. Front Bioeng Biotechnol. (2023) 11:1138596. doi: 10.3389/fbioe.2023.1138596, PMID: 36873375 PMC9978118

[94] Han J Lim J Wang CJ Han JH Shin HE Kim SN . Lipid nanoparticle-based mrna delivery systems for cancer immunotherapy. Nano Converg. (2023) 10:36. doi: 10.1186/s40580-023-00385-3, PMID: 37550567 PMC10406775

