# Supplementary material for: The multifaceted roles and diagnostic-therapeutic potential of LINC01410 in malignant tumors and non-malignant disorders
Source: Front Immunol. 2025 Dec 15;16:1588816. doi: 10.3389/fimmu.2025.1588816 (PMC12745413; doi:10.3389/fimmu.2025.1588816)
Supplement: Supplementary file 1 [file Table1.docx]

**Appendix**

| **Abbreviation** | **Full Name or Explanation** | **Abbreviation** | **Full Name or Explanation** |
| --- | --- | --- | --- |
| **OS** | osteosarcoma | **BC** | bladder cancer |
| **TC,** | thyroid cancer | **CC** | cervical cancer |
| **EC** | endometrial cancer | **ESCC** | esophageal squamous cell carcinoma |
| **GBM** | glioblastoma | **CCA** | cholangiocarcinoma |
| **NB** | neuroblastoma | **CRC** | colorectal cancer |
| **GBC** | gallbladder cancer | **NSCLC** | Non-Small Cell Lung Cancer |
| **DN** | Diabetic nephropathy | **PE** | Preeclampsia |
| **ceRNAs** | competitive endogenous RNAs | **ncRNAs** | Non-coding RNAs |
| **EMT** | epithelial-to-mesenchymal transition | **TMZ** | temozolomide |
| **CAFs** | cancer-associated fibroblasts | **LNM** | lymph node metastasis |
| **LD** | lipid droplets | **FASN** | fatty acid synthase |
| **FA** | fatty acid | **AUC** | Area Under the Curve |
| **STAT5** | Signal Transducer and Activator of Transcription 5 | **METTL3** | Methyltransferase Like 3 |
| **M6A** | N6-methyladenosine | **FASL** | Fas Ligand |
| **SMAD** | SMA-Mothers against decapentaplegic | **FOXM1** | Forkhead box M1 |
| **CHD7** | Chromodomain Helicase DNA Binding Protein 7 | **PKM2** | Pyruvate Kinase M2 |
| **HK2** | Hexokinase 2 | **NDRG3** | N-myc Downstream-Regulated Gene 3 |
| **HMGA2** | High Mobility Group AT-hook 2 | **WEE1** | WEE1 G2 Checkpoint Kinase |
| **VOPP1** | Viral Oncogene Overexpressed Protein 1 | **Snail1** | SNAIL Family Transcriptional Repressor 1 |
| **AGEs**  **MAFB** | Advanced Glycation End-products  MAF BZIP Transcription Factor B | **VEGF**  **PCNA** | Vascular Endothelial Growth Factor  cell nuclear antigen |
